# A multimodal vision foundation model for clinical dermatology

**DOI:** 10.1038/s41591-025-03747-y

**Published:** 2025-06-06

**Authors:** Siyuan Yan, Zhen Yu, Clare Primiero, Cristina Vico-Alonso, Zhonghua Wang, Litao Yang, Philipp Tschandl, Ming Hu, Lie Ju, Gin Tan, Vincent Tang, Aik Beng Ng, David Powell, Paul Bonnington, Simon See, Elisabetta Magnaterra, Peter Ferguson, Jennifer Nguyen, Pascale Guitera, Jose Banuls, Monika Janda, Victoria Mar, Harald Kittler, H. Peter Soyer, Zongyuan Ge

**Affiliations:** 1 AIM for Health Lab, Faculty of Information Technology, Monash University, Melbourne, Victoria Australia; 2 Faculty of Engineering, Monash University, Melbourne, Victoria Australia; 3 Frazer Institute, The University of Queensland, Dermatology Research Centre, Brisbane, Queensland Australia; 4 Victorian Melanoma Service, Alfred Hospital, Melbourne, Victoria Australia; 5 Department of Dermatology, Medical University of Vienna, Vienna, Austria; 6 eResearch Centre, Monash University, Melbourne, Victoria Australia; 7 NVIDIA AI Technology Center, Singapore, Singapore; 8 The University of Queensland, Brisbane, Queensland Australia; 9 Section of Dermatology, Department of Health Sciences, University of Florence, Florence, Italy; 10 Melanoma Institute Australia, Sydney, New South Wales Australia; 11 Tissue Pathology and Diagnostic Oncology, Royal Prince Alfred Hospital and NSW Health Pathology, Sydney, New South Wales Australia; 12 Sydney Melanoma Diagnostic Centre, Royal Prince Alfred Hospital, Sydney, New South Wales Australia; 13 Department of Dermatology, Hospital General Universitario de Alicante, ISABIAL, Alicante, Spain; 14 Department of Clinical Medicine, Universidad Miguel Hernández, Sant Joan D’Alacant, Spain; 15 Centre for Health Services Research, Faculty of Medicine, The University of Queensland, Brisbane, Queensland Australia; 16 School of Public Health and Preventive Medicine, Monash University, Melbourne, Victoria Australia; 17 Dermatology Department, Princess Alexandra Hospital, Brisbane, Queensland Australia; 18 Airdoc-Monash Research, Monash University, Melbourne, Victoria Australia

**Keywords:** Machine learning, Skin cancer, Diagnosis, Imaging, Computational models

## Abstract

Diagnosing and treating skin diseases require advanced visual skills across domains and the ability to synthesize information from multiple imaging modalities. While current deep learning models excel at specific tasks such as skin cancer diagnosis from dermoscopic images, they struggle to meet the complex, multimodal requirements of clinical practice. Here we introduce PanDerm, a multimodal dermatology foundation model pretrained through self-supervised learning on over 2 million real-world skin disease images from 11 clinical institutions across 4 imaging modalities. We evaluated PanDerm on 28 diverse benchmarks, including skin cancer screening, risk stratification, differential diagnosis of common and rare skin conditions, lesion segmentation, longitudinal monitoring, and metastasis prediction and prognosis. PanDerm achieved state-of-the-art performance across all evaluated tasks, often outperforming existing models when using only 10% of labeled data. We conducted three reader studies to assess PanDerm’s potential clinical utility. PanDerm outperformed clinicians by 10.2% in early-stage melanoma detection through longitudinal analysis, improved clinicians’ skin cancer diagnostic accuracy by 11% on dermoscopy images and enhanced nondermatologist healthcare providers’ differential diagnosis by 16.5% across 128 skin conditions on clinical photographs. These results show PanDerm’s potential to improve patient care across diverse clinical scenarios and serve as a model for developing multimodal foundation models in other medical specialties, potentially accelerating the integration of artificial intelligence support in healthcare.

## Abstract (web summary)

Trained on large and multicenter datasets with different imaging modalities, a foundation model is shown to have strong performance on the full spectrum of clinically relevant tasks and to increase user accuracy in diagnostic tasks.

## Main

There is a pressing need to fully harness the potential of artificial intelligence (AI) in diagnosing and managing skin diseases. Although deep learning has shown remarkable performance, often matching or surpassing dermatologists, current AI models for dermatology remain limited to isolated tasks, such as diagnosing skin cancer from dermoscopic images^1^. These models struggle to integrate various data types and imaging modalities, reducing their utility in different real-world clinical settings. Dermatology, like internal medicine, is inherently complex, encompassing a broad spectrum of conditions from common dermatoses to life-threatening malignancies, necessitating a comprehensive, patient-centered approach that integrates various clinical workflows.

In clinical practice, diagnosing and treating skin conditions involves a range of tasks, including total-body skin cancer detection and risk assessment^2–5^, differential diagnosis of hundreds of dermatological conditions such as inflammatory dermatoses and pigmentary disorders^6^, multimodal image analysis^7,8^, pathology interpretation^9,10^, monitoring lesion changes^11,12^ and predicting outcomes^13,14^. The absence of integrated AI solutions capable of supporting these various workflows currently hampers the practical impact of AI in dermatology. Recent advances in foundation models have emerged as a promising direction to address this challenge^15,16^.

Foundation models are large-scale neural networks pretrained on vast, diverse data using self-supervised learning techniques, often leveraging weakly labeled or unlabeled data^17–19^. Built on rich knowledge representations, these models have shown impressive performance across medical fields such as ophthalmology^20^, radiology^21^ and pathology^22–25^. Through comprehensive pretraining on large and diverse data, these models develop versatile representations that can effectively adapt to various clinical scenarios, outperforming previous deep learning models in downstream tasks. Their strong feature representations also enable data-efficient applications^26,27^, requiring fewer labeled samples, which is particularly crucial for medical domains in which expert-annotated data are often limited.

However, developing effective foundation models for dermatology presents unique challenges. The performance of foundation models is inherently linked to the scale of their parameters and training data^28–30^. In general computer vision, foundation models are pretrained on massive datasets such as ImageNet^31^ or JFT-300M (ref. ^32^) and most existing dermatology AI models still rely on these models for downstream adaptation. Some efforts have focused on self-supervised learning specifically for dermatology using public datasets^33,34^ or web-sourced skin images^35^. However, these approaches are often limited by dataset size, diversity or the lack of real patient data. Moreover, while recent advances in medical foundation models have shown promise in various specialties, they cannot fully address dermatology’s unique needs. Specialty-specific foundation models^20,21,23^ typically focus on single imaging modalities, while general biomedical models, despite their broad scope, struggle with domain-specific data scarcity and integrating heterogeneous modalities for comprehensive clinical analysis.

**Fig. 1:**
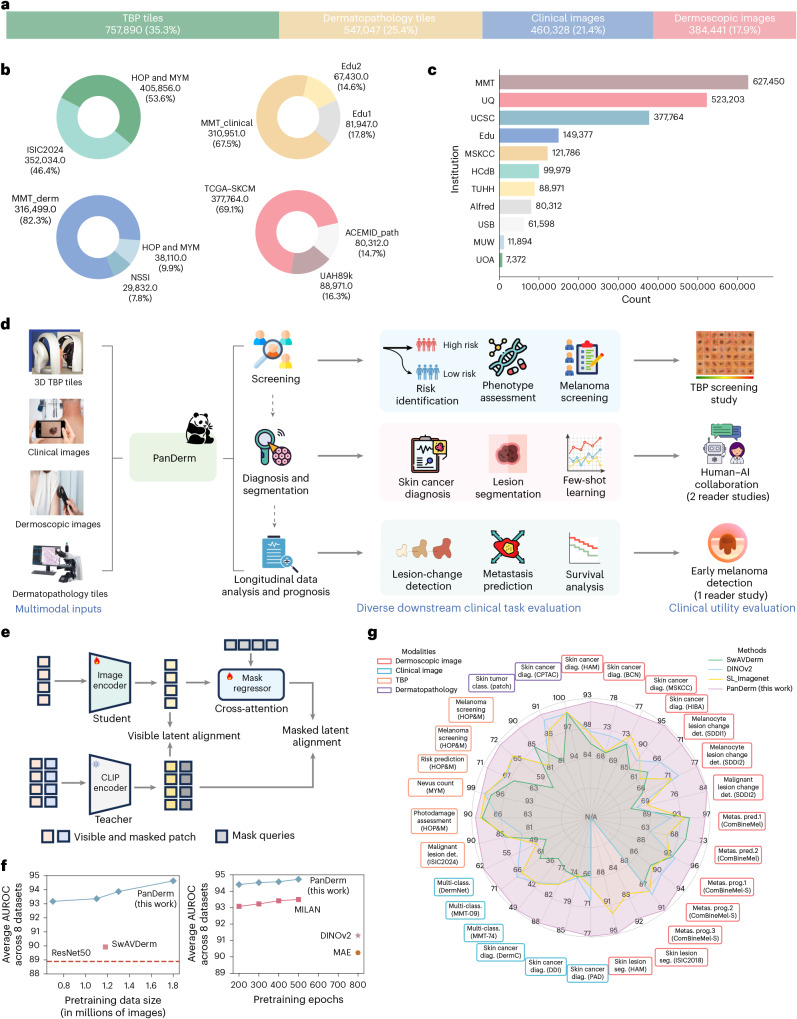
Overview of this study. **a**–**c**, Pretraining dataset: 2.1 million dermatological images from 11 clinical sources across 4 modalities, shown by modality (**a**), source (**b**) and institution (**c**). **d**, PanDerm interprets multiple imaging modalities for various dermatology tasks, evaluated in real-world melanoma screening and three reader studies. Image types include dermatopathology (microscopic biopsy specimens), clinical (wide-field lesion and surrounding skin), dermoscopic (close-up dermoscope images) and TBP tiles (lesion crops). **e**, Architecture: ViT-large encoder, regressor and CLIP-based teacher model, with representation reconstruction and CLIP latent alignment objectives. **f**, Performance versus pretraining data size and epochs (average AUROC on 8 benchmarks) compared with alternative strategies. **g**, PanDerm outperforms existing models on 28 evaluation datasets across 4 modalities. All icons in **d** are from Flaticon.com, except for the risk stratification, lesion change detection and survival analysis icons, which are from Microsoft PowerPoint.

Here we introduce PanDerm, a general-purpose, multimodal dermatology foundation model. Uniquely designed to integrate multiple imaging modalities, PanDerm is pretrained on over 2 million images sourced from 11 institutions across multiple countries, covering 4 imaging modalities spanning diverse dermatological conditions (Fig. 1a–c). In the pretraining stage, PanDerm uses the masked latent modeling and contrastive language-image pre-training (CLIP)^36^ feature alignment for self-supervised learning (Fig. 1e and Methods), showing superior data scalability and training efficiency compared with existing self-supervised algorithms (Fig. 1f). The model achieves unified representation learning across total-body photography (TBP) and clinical, dermoscopic and dermatopathology images, enabling comprehensive patient analysis throughout diverse clinical workflows (Fig. 1d).

We systematically evaluate PanDerm across 28 benchmarks (Fig. 1g), covering a diverse array of clinical tasks, including screening, risk stratification, phenotype assessment, nevus counting, longitudinal monitoring, lesion change detection, diagnosis of both common and rare skin conditions and skin lesion segmentation, as well as recurrence prediction and prognosis. PanDerm achieves state-of-the-art performance on all tasks, often using only 5–10% of the labeled training data typically required. Through three reader studies, we show that this unified multimodal approach outperforms clinicians in early-stage melanoma detection, enhances clinicians’ diagnostic accuracy in skin cancer diagnosis and supports nonspecialist healthcare providers in the differential diagnosis of diverse skin conditions. These findings highlight the potential of specialty-specific foundation models to advance medical practice by integrating diverse modalities, with broader implications for AI development across healthcare specialties.

## Results

### Ablation studies and training strategy comparisons

To evaluate PanDerm’s effectiveness, we conducted systematic analyses examining how model performance scales with training data and computational resources (datasets described in Supplementary Table 1). First, compared with existing dermatology-specific models, PanDerm showed strong scalability as training data increased from 0.8 to 1.8 million skin images (Fig. 1f, left). Notably, it achieved superior performance to SwAVDerm^35^, a leading dermatology self-supervised learning model, using 33% less training data. When compared with other self-supervised training techniques, PanDerm showed remarkable computational efficiency, requiring only 200 training epochs to achieve the best performance, compared with 500–800 epochs needed by leading methods such as MILAN^37^, DINOv2 (ref. ^38^) and MAE^19^ (Fig. 1f, right). Furthermore, PanDerm also surpassed vision-language models such as CLIP^36^, MONET^39^ and biomedical-specific CLIP (BiomedCLIP)^40^ in benchmark evaluations (Supplementary Table 1), while showing emergent capabilities in dermatology similar to those of DINOv2 in natural images, with linear probing performance comparable to full-parameter fine-tuning (Supplementary Table 2). When evaluated against generalist biomedical foundation models, PanDerm showed substantial advantages across different dermatological tasks. Compared with a representative model in this category, BiomedGPT^41^, PanDerm showed 20.9% better area under the receiver operating characteristic curve (AUROC) in melanoma detection, 34.7% higher weighted F1 score in differentiating between skin conditions and 19.6% improved weighted F1 in analyzing microscopic skin tissue images (Extended Data Table 1). Even using computationally efficient methods, PanDerm maintained its advantages, outperforming both linear-probe and fine-tune versions of BiomedGPT by 14.3% and 5.1%, respectively, in linear probing (Supplementary Table 3). On the basis of these promising results, we expanded our evaluation to compare PanDerm with three representative AI models: SL-Imagenet^31^ and DINOv2 (ref. ^38^) (both widely used foundation models pretrained on natural images with a ViT-Large^42^ backbone), and SwAVDerm^35^ (a self-supervised model pretrained on a large skin image dataset from search engines).

### Diagnostic performance and generalization ability across datasets

**Fig. 2:**
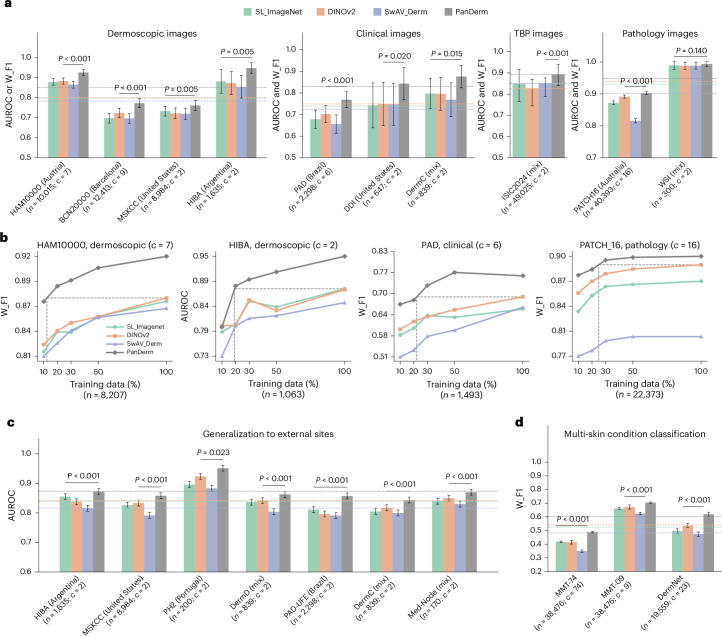
PanDerm’s versatile capacity in diverse diagnosis tasks. **a**, Performance comparison of PanDerm versus other pretrained models on 10 pigmented skin lesion datasets across multiple centers and modalities. *n*, data size; *c*, class number. Metrics: AUROC for binary class (*c* = 2) and W_F1 score for multi-class (*c* > 2) datasets. The dashed lines indicate the average model performance across datasets. **b**, Comparison between PanDerm and other pretrained models in label efficiency generalization on four representative datasets, showing performance at various training data percentages. The vertical dashed lines indicate the data quantity needed for PanDerm to match existing model performance. **c**, External validation for melanoma diagnosis across 7 datasets. **d**, Performance evaluation of general skin condition classification (up to 74 classes) using clinical images. The error bars in **a**, **c** and **d** show 95% CIs; bar centers in **a**, **c** and **d** represent mean values; dots in **b** represent mean values. Estimates were computed using nonparametric bootstrapping with 1,000 bootstrap replicates. *P* values were calculated using a two-sided *t*-test.

**Extended Data Fig. 1:**
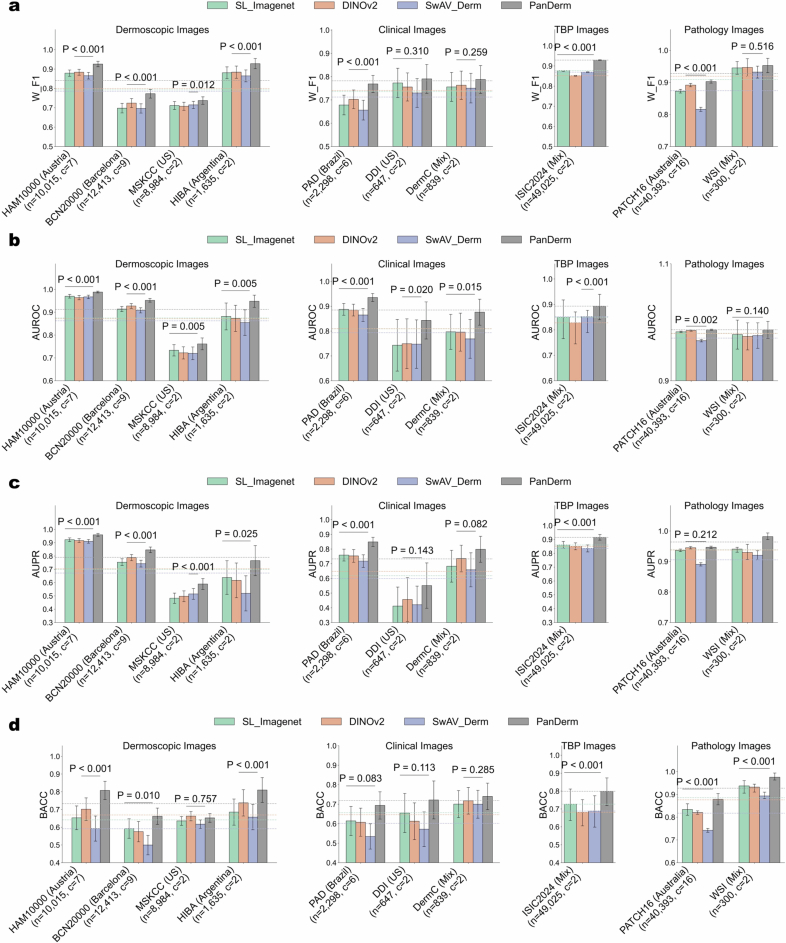
Performance of PanDerm versus other pretrained models on 10 pigmented skin lesion datasets across multiple centers and modalities. **a**. Performances are measured by weighted F1 (W F1). **b**. Performances are measured by AUROC. **c**. Performances are measured by AUPR. **d**. Perfor- mances are measured by BACC. n: data size, c: class number. Dashed lines show the average performance of each model across different datasets. Estimates were computed using nonparametric bootstrapping with 1000 bootstrap replicates. P-values calculated using a two-sided t-test. Error bar, 95% CIs; bar centers, means.

We systematically evaluated PanDerm diagnostic performance across 10 public datasets from 4 imaging modalities and 7 international sites (Fig. 2a). These datasets covered multi-class classification of pigmented neoplastic lesions and binary melanoma diagnosis tasks. PanDerm consistently outperformed all other models, achieving significant improvements on 9 of 10 datasets, with average gains of 5.1%, 8.0%, 4.2% and 0.9% on dermoscopic, clinical, TBP and pathology datasets, respectively (Fig. 2a). On representative dermoscopy and clinical benchmarks such as HAM10000 (ref. ^34^) and PAD-UFES-20 (ref. ^43^), PanDerm surpassed the next-best models by 4.7% (*P* < 0.001) and 9.0% (*P* < 0.001), respectively (Fig. 2a, Supplementary Table 4 and Extended Data Fig. 1).

**Extended Data Fig. 2:**
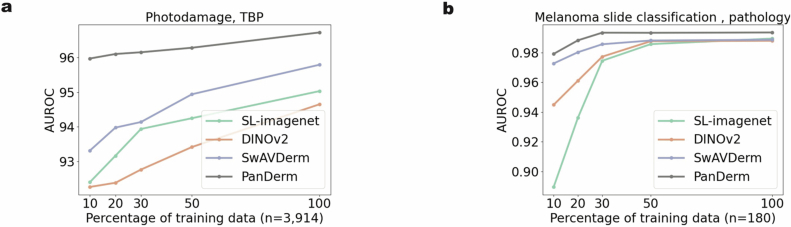
Label efficiency generalization results on additional tasks. **a**. Label efficiency analysis for photodamage risk assessment using Total Body Photography (TBP) images. Results demonstrate model performance with limited labeled data available. PanDerm outperformed the second-best models using only 10% of labeled images. **b**. Label efficiency analysis for melanoma classification using whole slide dermatopathology images. Results illustrate model performance with limited labeled data. PanDerm surpassed the second-best models using less than 30% of labeled images.

PanDerm showed strong performance even with limited training data, achieving comparable results to other models while using only 10–30% of the labeled images (Fig. 2b and Supplementary Tables 5–10). Additional results for other tasks are presented in Extended Data Fig. 2. To test PanDerm’s generalization applicability, we evaluated its performance on melanoma diagnosis using images from seven external medical centers, representing patient populations different from the training data. PanDerm showed significant superiority over all pretrained models, achieving higher AUROC scores across all external datasets (Fig. 2c). Notably, it maintained high performance even on clinical photographs that were not used during training, with AUROC gains of 4.0%, 2.6% and 2.1% on the three external clinical datasets (all *P* < 0.001).

Beyond skin cancer diagnosis, we evaluated PanDerm’s ability to diagnose a broader range of skin conditions commonly seen in clinical practice. We tested on three complementary datasets: the public DermNet dataset^44^ covering 23 common conditions, along with two internal datasets (MMT-09 with 9 conditions and MMT-74 with 74 conditions) comprising 38,476 clinical images across 9 broad and 74 fine-grained skin conditions. These datasets comprehensively cover inflammatory diseases, infections, various types of skin tumors and other frequently encountered skin problems. As shown in Fig. 2d, PanDerm achieved weighted F1 improvements of 3.2%, 7.1% and 8.2% on MMT-09, DermNet and MMT-74, respectively, compared with the next-best models (all *P* < 0.001). PanDerm’s advantage grew larger as the number of conditions increased, showing its strong capability to handle complex, multi-disease scenarios. PanDerm also outperformed all other pretrained models on all metrics across the three datasets (all *P* < 0.001; Supplementary Table 11). In the DermNet dataset, PanDerm exceeded the next-best model’s area under the precision–recall curve (AUPR) by 14.7%. Further details on the experimental setup, datasets and metrics are provided in Methods.

### Short-term lesion change detection in sequential dermoscopic images

**Fig. 3:**
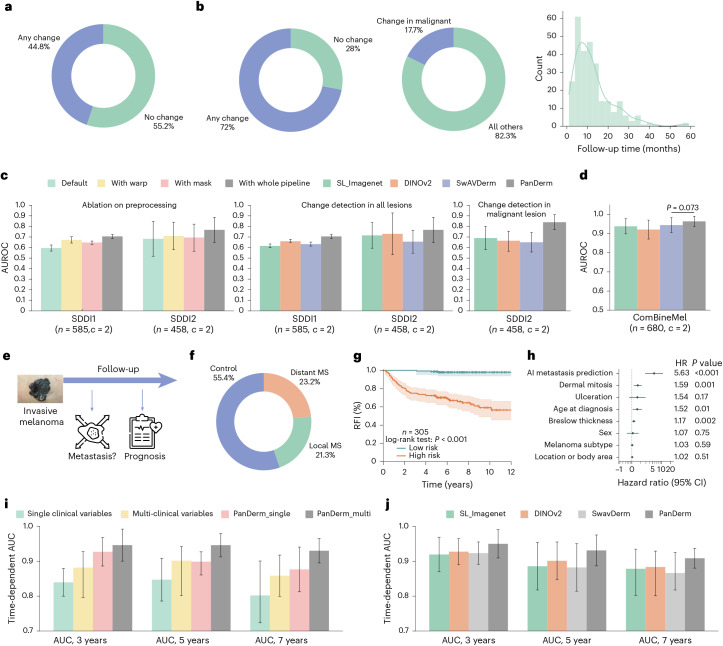
Short-term lesion change detection and metastasis prognosis results. **a**, SDDI1 dataset (*n* = 585 dermoscopic images) statistics: ratio of changed lesions, ratio of changed malignant lesions during follow-up, and follow-up time distribution. **b**, Ratio of changed lesions in the SDDI2 dataset (*n* = 458 dermoscopic images). **c**, Ablation study on preprocessing methods using SDDI1 and SDDI2 ‘Default’ (direct input), ‘With warp’ (registration only), ‘With mask’ (lesion segmentation) and ‘With whole pipeline’ (complete preprocessing as in Extended Data Fig. 3). For change detection in SDDI1 and SDDI2, all models were evaluated using the whole preprocessing pipeline. **d**, Performance of binary metastasis prediction (control versus metastasis) in ComBineMel (*n* = 680 dermoscopic images) by AUROC. **e**, Scheme of PanDerm for melanoma metastasis and prognosis prediction. MS, metastasis. **f**, Distribution of metastasis types in the ComBineMel dataset (*n* = 680 dermoscopic images). **g**, Kaplan–Meier curves for the RFI in invasive melanoma patients (ComBineMel (*n* = 305 patients)), stratified by PanDerm prediction scores. **h**, Forest plot of HRs for PanDerm; stratified groups in invasive melanoma patients. **i**, Time-dependent AUC of PanDerm versus clinical variable score combinations in ComBineMel. **j**, Time-dependent AUC comparison of PanDerm and other pretrained models in ComBineMel. The error bars in **c**, **d**, **i** and **j** and error bands in **g** show 95% CIs; the bar centers indicate means. All estimates were derived from fivefold cross-validation. *P* values in **d** were derived from two-sided *t*-tests and those in **h** from Wald tests within Cox proportional hazards models. Icons in **e** from Flaticon.com.

**Extended Data Fig. 3:**
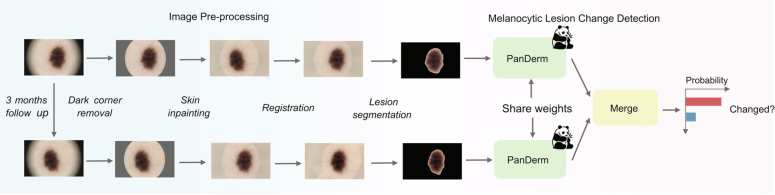
Longitudinal dermoscopic image-based lesion change detection using PanDerm. For comparing subtle changes in paired lesions during short-term follow-up (for example, 3 months), images undergo dark corner detection and removal, skin inpainting, registration, and lesion segmentation. This allows models to focus on subtle differences between lesions at different time points. Panda icon from Flaticon.com.

Monitoring suspicious melanocytic lesions over a 3-month period is a widely accepted procedure for early melanoma detection, as changes often prompt excision to rule out melanoma, while stability can be reassuring^12^. We evaluated PanDerm’s ability to detect subtle changes in lesions over time by analyzing pairs of sequential dermoscopic images. To ensure accurate comparison despite variations in imaging conditions, we developed a comprehensive image-processing system that standardizes image quality and alignment (Extended Data Fig. 3). This processing system, combined with PanDerm’s advanced lesion change detection capabilities^45^, significantly improved change detection accuracy from 0.596 (95% confidence interval (CI) 0.567–0.624) to 0.706 (95% CI 0.686–0.725) in sequential digital dermoscopic imaging data (SDDI1) (Fig. 3a,c) (*P* < 0.001) and from 0.683 (95% CI 0.517–0.894) to 0.767 (95% CI 0.649–0.886) in SDDI2 (Fig. 3b,c, left) (*P* < 0.001). Using the optimized pipeline for all models, PanDerm achieved AUROC improvements of 4.3% in SDDI1 (*P* < 0.001) and 3.7% in SDDI2 over the next-best model (Fig. 3c, middle). For lesions later diagnosed as malignant, PanDerm achieved an AUROC of 0.840 (95% CI 0.769–0.911), surpassing the next-best model by 15.0% (*P* *<* 0.01) (Fig. 3c, right). Further details on the lesion change detection method and dataset details are provided in Methods and Supplementary Tables 12–14.

### Melanoma metastasis prediction and survival analysis

We explored PanDerm’s potential to predict melanoma progression from dermoscopic images, an emerging approach that could provide valuable prognostic information at the time of diagnosis^13,14,46^ (Fig. 3e). We evaluated this capability using 680 dermoscopic images from 370 patients with invasive primary melanoma across multiple international centers (Fig. 3f). PanDerm showed exceptional accuracy in distinguishing melanomas likely to metastasize, achieving an AUROC of 0.964 (95% CI 0.937–0.991), surpassing the next-best model by 2.0% (*P* = 0.073) (Fig. 3d). It also showed strong capability in differentiating between local and distant metastases, outperforming existing methods by 2.8% (*P* < 0.05) in the weighted F1 score (Supplementary Table 15).

To validate PanDerm’s clinical utility for patient risk stratification, we conducted survival analyses using Kaplan–Meier analysis and Cox proportional hazards regression. Patients classified as high risk by PanDerm showed significantly shorter recurrence-free intervals (RFIs) compared with those in the low-risk group (hazard ratio (HR) 5.63; 95% CI 2.87–11.02, *P* < 0.001) (Fig. 3g). When compared alongside standard clinical risk factors—including sex, age, Breslow thickness, ulceration, dermal mitosis, location and melanoma subtype—PanDerm’s predictions emerged as the strongest indicator of recurrence risk in multivariate Cox regression (Fig. 3h). It maintained high predictive accuracy over extended follow-up periods, with time-dependent areas under the curve (AUCs) of 0.950 (95% CI 0.910–0.991), 0.931 (95% CI 0.887–0.976) and 0.909 (95% CI 0.880–0.937) at 3 years, 5 years and 7 years, exceeding multi-clinical variables by 6.8%, 2.9% and 5.0%, respectively (Fig. 3i). Combining PanDerm’s predictions with clinical factors further improved long-term prognostic accuracy in AUCs at 5 years and 7 years. PanDerm also consistently outperformed other AI approaches (Fig. 3j), showing improvements of 2.3%, 3.0% and 2.5% at 3 years, 5 years and 7 years, respectively. Further details are provided in Methods and Supplementary Tables 16 and 17.

### Risk assessment and malignant lesion screening using TBP

**Fig. 4:**
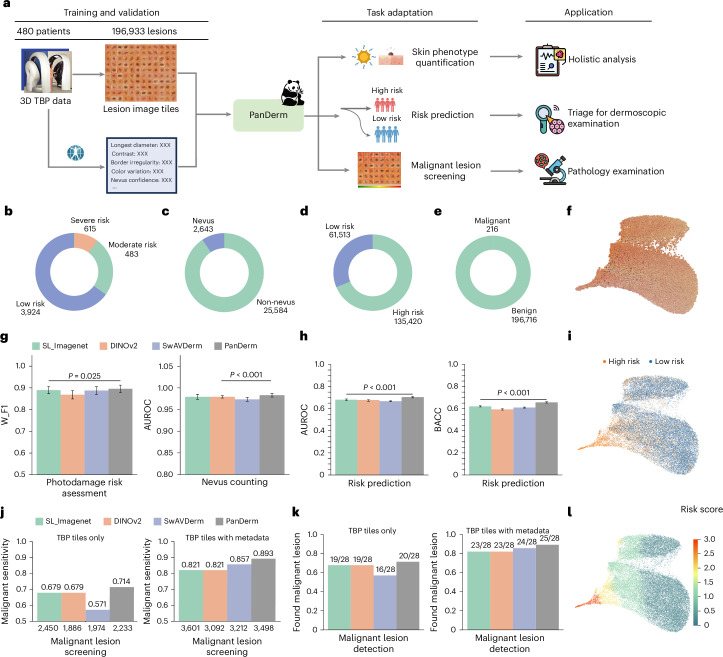
Skin phenotype assessment and malignant lesion screening using TBP. **a**, Illustration of PanDerm processing multimodal TBP data for skin phenotype quantification, risk prediction and malignant lesion screening. **b**,**c**, Class distribution of skin phenotype quantification for photodamage risk assessment (*n* = 5,022 TBP tiles) (**b**) and nevus counting (*n* = 28,227 TBP tiles) (**c**) in datasets. **d**,**e**, Class distribution of risk prediction (**d**) and benign and malignant lesions (*n* = 196,933 TBP tiles) (**e**). **g**, Photodamage risk assessment and nevus counting performance by W_F1 and AUROC. **h**, Risk prediction performance by AUROC and BACC. Error bars in **g** and **h** show 95% CIs; bar centers represent mean values. Estimates were computed with nonparametric bootstrapping using 1,000 bootstrap replicates. *P* values were calculated with a two-sided *t*-test. **j**, Malignant lesion screening performance by sensitivity. Left: using only TBP data; right: integrating measurement information. The numbers below the bars indicate the recommended suspicious lesion count. **k**, Number of malignant lesions detected in the test set. **f**, UMAP plot of PanDerm screening results for test lesions. **i**, UMAP plot of human screening results for test lesions. **l**, UMAP plot of PanDerm risk prediction results for test lesions. All icons in **a** are from Flaticon.com, except the risk prediction icon, which is from Microsoft PowerPoint.

We next evaluated PanDerm’s capability in analyzing whole-body imaging (TBP)^2,3,47^ (Fig. 4a). Unlike close-up imaging of individual lesions, TBP enables comprehensive patient-level analysis, particularly for critical melanoma risk factors such as photodamage and nevus count^4,5,48^. In a cohort of 480 patients with 196,933 lesions from Australia, PanDerm achieved a weighted F1 score of 0.896 (95% CI 0.879–0.913) for photodamage assessment and an AUROC of 0.983 (95% CI 0.979–0.987) for nevus counting, surpassing all other models (*P* *<* 0.05 and *P* *<* 0.001, respectively; Fig. 4b,c,g). Even with limited training data (10% of the full dataset), PanDerm maintained superior performance (Extended Data Fig. 2). In lesion-specific risk stratification, PanDerm also ranked first with an AUROC of 0.705 (95% CI 0.698–0.712) and balanced accuracy (BACC) of 0.657 (95% CI 0.6513–0.663), with all results statistically significant (*P* < 0.001; Fig. 4d,h).

In a clinical validation study, PanDerm effectively identified malignant lesions among a large number of benign ones (216 malignant versus 197,716 benign lesions) from the high-risk melanoma of patients (HOP) study^49^ and mind your model (MYM) study^50^ cohort (Fig. 4e). Using TBP images alone, PanDerm achieved a sensitivity of 0.893, outperforming the next-best model by 4.2% (Fig. 4j, left). When clinical measurements were available for all models, PanDerm maintained its advantage with a 3.5% higher sensitivity (Fig. 4j, right), reaching a sensitivity of 0.893. Significantly, it detected malignant lesions in 79 out of 80 patients while reducing unnecessary examinations by 60.8% compared with melanographers (3,498 versus 8,913 lesions recommended for detailed examination) (Fig. 4j,k and Supplementary Table 18).

**Extended Data Fig. 4:**
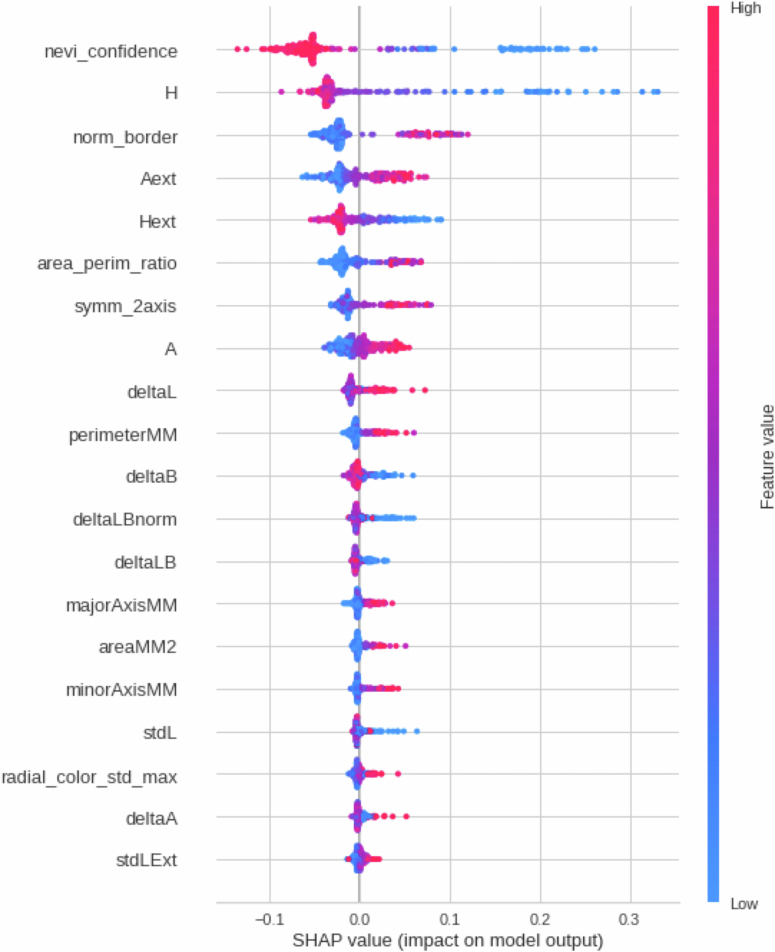
SHAP (SHapley Additive exPlanations) value plot. It shows the impact of various measurement variables captured by the 3D TBP machine on the model output. The plot displays the relative importance and directional influence of each feature, with colors indicating high (red) to low (blue) feature values, and the x-axis representing the SHAP value or impact on the model’s prediction. Features are ordered by their overall importance, with ‘nevi confidence’ having the highest impact and ‘stdLExt’ the lowest.

We observed that PanDerm’s analysis approach aligned well with established clinical practice, particularly the ‘ugly duckling’ (UD) concept^51^ of identifying atypical lesions through comparison with a patient’s other lesions. This was shown through UMAP visualization (Fig. 4f), where PanDerm’s feature effectively separated suspicious lesions. The clustering patterns in PanDerm’s risk assessment (Fig. 4l) showed correspondence closely with human screening patterns (Fig. 4i), illustrating its exceptional performance in malignant lesion screening. Additional details are provided in Methods, Supplementary Tables 18–21 and Extended Data Fig. 4.

### Skin lesion segmentation

**Extended Data Fig. 5:**
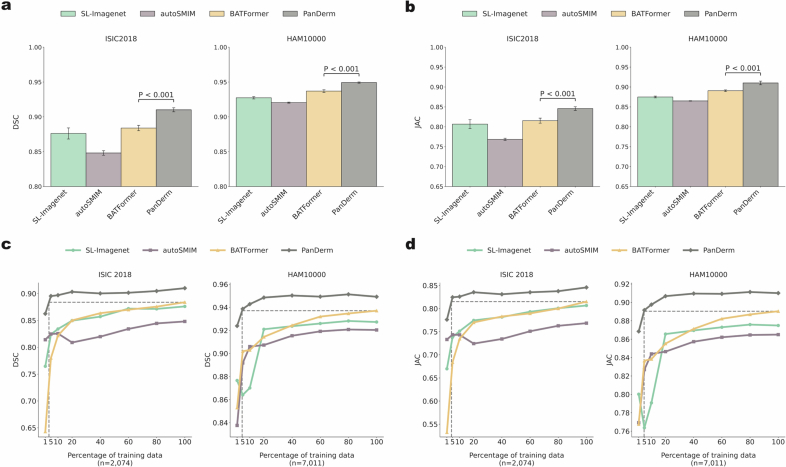
Quantitative skin lesion segmentation results. **a**, **b**. Segmentation performance measured by dice score (DSC) and Jaccard index (JAC) for PanDerm and baseline models on ISIC2018 (n=2,074 dermoscopic images) and HAM10000 (n=7,011 dermoscopic images) datasets. **c**, **d**. Label efficiency generalization performance for PanDerm and baselines, showing mean DSC and JAC on ISIC2018 and HAM10000 datasets. Error bars in **a**, **b** indicate 95% confidence intervals; bar centers represent mean values; points in **c**, **d** denote mean values. All estimates are derived from five replicas with different seeds. Statistical significance was assessed using two-sided t-tests.

**Extended Data Fig. 6:**
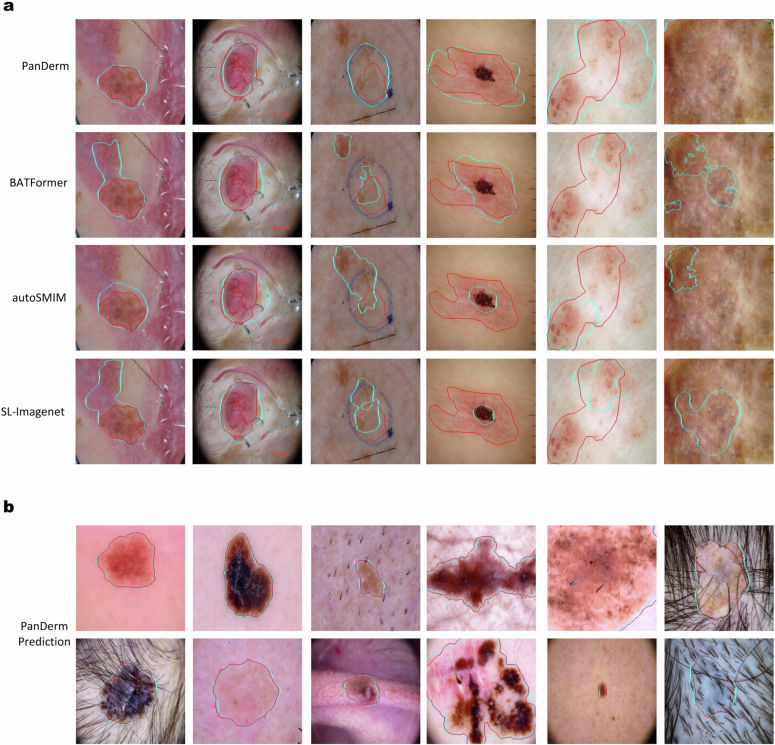
Qualitative skin lesion segmentation results. **a**. Comparison of PanDerm against baseline models on challenging examples from HAM10000. Red contours indicate ground truth masks, while cyan contours show model predictions. **b**. PanDerm segmentation results on a random selection of images from HAM10000.

We evaluated PanDerm’s performance on skin lesion segmentation using the ISIC2018 (ref. ^52^) and HAM10000 (ref. ^34^) datasets. Compared with existing methods including SL-Imagenet, autoSMIM^33^ and BATFormer^33^, PanDerm achieved significantly higher performance, surpassing the next best by 3.1% and 1.9% in the Jaccard index on both datasets (*P* *<* 0.001; Extended Data Fig. 5a,b). PanDerm’s performance was particularly noteworthy in label-limited scenarios, matching the next-best model while using only 5% of the training data (104 and 350 images for ISIC2018 and HAM10000, respectively; Extended Data Fig. 5c,d). When compared with MedSAM^53^, a medical image segmentation foundation model, PanDerm showed slightly better accuracy (0.5% improvement, *P* = 0.025 and 0.112; Supplementary Table 22). This is particularly impressive as PanDerm achieves this performance without specialized training for image segmentation. In addition, PanDerm offers practical advantages in clinical settings, processing images about four to five times faster than MedSAM while using less computational resources (Supplementary Table 23). Visual examples and detailed performance metrics are provided in Extended Data Fig. 6 and Supplementary Tables 22–25.

### Reader studies

To assess PanDerm’s clinical applicability, we conducted three reader studies evaluating its capabilities across different aspects and modalities of dermatological diagnosis, as follows.

#### Early melanoma detection compared with clinicians

**Extended Data Fig. 7:**
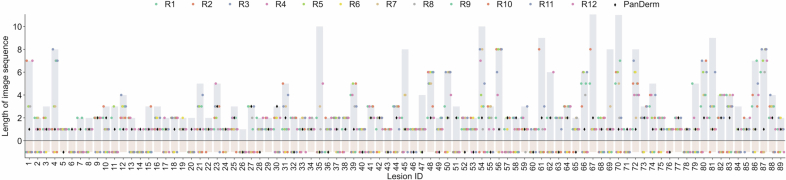
Early melanoma detection results (reader study 1). Comparing PanDerm to 12 clinicians (7 experienced dermatologists, 5 dermatology residents). X-axis: 89 melanoma lesion IDs; Y-axis: lesion image sequence length. Points on the histogram represent the initial time points of correct melanoma diagnoses. Points below y=0 correspond to melanoma lesions undetected throughout the sequence.

To examine PanDerm’s capability in early melanoma detection, we compared it with 12 human reviewers (7 experienced dermatologists and 5 dermatologist trainees) using sequential dermoscopic images from Alfred Hospital^54^, featuring multiple follow-up images of the same lesions over time. The study evaluated two key aspects: overall diagnostic accuracy and early melanoma detection capability. In terms of overall accuracy, PanDerm outperformed the average human reviewer by 10.2% and surpassed the best-performing human by 3.6%. For early detection, we assessed the time point of the first suspicious changes detected in sequential images relative to clinical diagnosis and biopsy confirmation. PanDerm showed superior ability in this challenging task, correctly identifying 77.5% (69 out of 89) of melanoma lesions at the first imaging time point, compared with only 32.6% (29 correct diagnoses) for human reviewers (Extended Data Fig. 7). Individual dots in the histograms represent the earliest correct diagnosis time points for both PanDerm and human reviewers, visualizing the comparative early detection performance.

#### Human–AI collaboration for skin cancer diagnosis

**Fig. 5:**
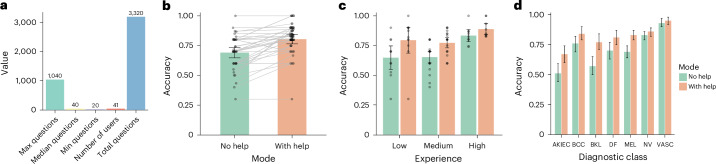
Performance of PanDerm in human–AI collaborative skin cancer diagnosis using dermoscopic images. **a**, Reader study overview: 41 users answered 3,320 questions on the ISIC2018 Task 3 test set (*n* = 1,511 images, 7 classes). **b**, Diagnostic accuracy comparison: without versus with PanDerm support (*P* < 0.001; two-sided paired *t*-test; *n* = 41 readers). **c**, Accuracy comparison without versus with PanDerm by experience level based on experience per experience: low (*n* = 11), medium (*n* = 21) and high (*n* = 9). **d**, Accuracy comparison without versus with PanDerm by diagnostic class based on readings per class: MEL (*n* = 332), BCC (*n* = 166), AKIEC (*n* = 166), BKL (*n* = 166), NV (*n* = 498), DF (*n* = 166) and VASC (*n* = 166). The error bars represent 95% CIs; bar centers represent means.

We evaluated PanDerm’s impact on clinicians’ diagnostic accuracy across seven pigmented lesion classes using dermoscopic images (Fig. 5a). The study included 41 clinicians with varying levels of competency who evaluated cases both with and without PanDerm’s multi-probability prediction support. PanDerm’s assistance significantly increased overall diagnostic accuracy from 0.69 (95% CI 0.65–0.73) to 0.80 (95% CI 0.76–0.84, *P* *<* 0.001; Fig. 5b). Notably, clinicians with lower competency levels showed the greatest improvement, with accuracy gains of 17% (*P* = 0.0082) for those with low competency and 12% (*P* < 0.001) for those with medium competency, while highly competent clinicians showed a 6% improvement (*P* = 0.039; Fig. 5c and Supplementary Table 26). Class-specific analysis revealed significant accuracy improvements in 4 of 7 lesion classes (*P* < 0.05; Fig. 5d and Supplementary Table 27). For melanoma diagnosis specifically, PanDerm enhanced clinician accuracy from 0.69 (95% CI 0.64–0.74) to 0.83 (95% CI 0.79–0.87, *P* *<* 0.001). In addition, PanDerm alone achieved diagnostic accuracy comparable to that of clinicians with PanDerm assistance (0.81 versus 0.80; *P* = 0.779).

#### Human–AI collaboration for 128 skin condition diagnoses

**Fig. 6:**
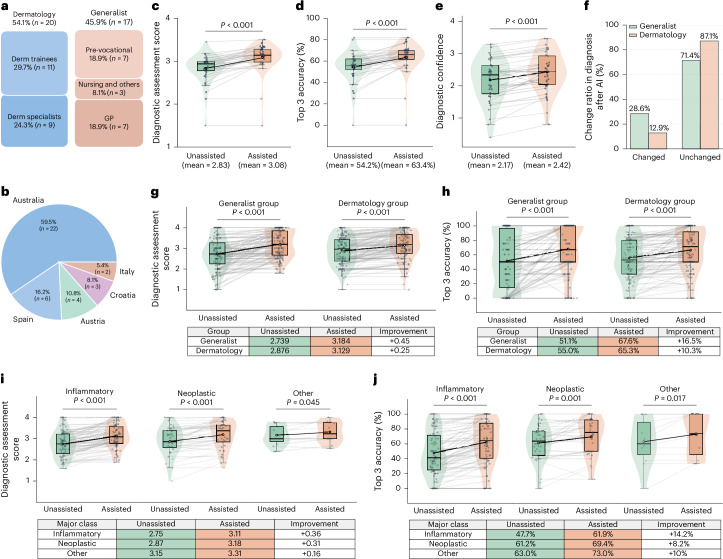
Performance of PanDerm in human–AI collaborative assessment of 128 skin conditions using clinical images. **a**, Reader demographics (*n* = 37 readers): dermatology group (*n* = 20 readers) including residents and specialists, and generalist group (*n* = 17 readers) including pre-vocational trainees, general practitioners, nurses and clinical trial assistants. Each reviewed up to 50 of 200 cases. **b**, Geographic distribution of readers. **c**–**e**, Reader-wise analysis (each data point represents one reader, *n* = 37 readers): comparisons without versus with PanDerm support for: top 1 diagnostic assessment score (1–4) (**c**), top 3 diagnostic accuracy (**d**) and diagnostic confidence score (1–4) (**e**). **f**, Diagnosis change ratio after PanDerm support by specialization group. **g**,**h**, Class-wise analysis (each data point represents one skin condition class): comparisons without versus with PanDerm support by specialization groups for the top 1 diagnostic assessment score (1–4) (**g**) and top 3 diagnostic accuracy (**h**) (*n* = 128 classes per group). **i**,**j**, Comparisons without versus with PanDerm support by disease category for the top 1 diagnostic assessment score (1–4) (**i**) and the top 3 diagnostic accuracy (**j**), stratified by inflammatory (*n* = 78 classes), neoplastic (*n* = 37 classes) and other (*n* = 13 classes) conditions. *P* values in **c**–**e** were calculated using two-sided paired *t*-test across readers, while *P* values in **g**–**j** were calculated using two-sided paired *t*-test across classes. In all the boxplots, the horizontal lines represent medians and the white dots represent means. The upper and lower box limits indicate the 1st and 3rd quartiles, with whiskers extending to 1.5 times the interquartile range. Error bars represent 95% CIs.

**Extended Data Fig. 8:**
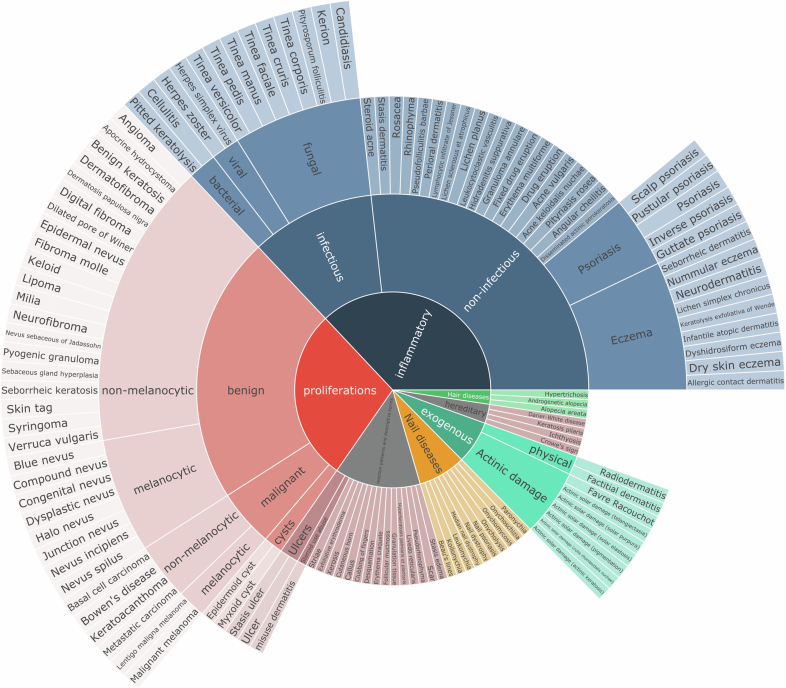
Sunburst plot of standard ontology on SD-128 dataset. Four experienced dermatologists collaboratively developed the standard ontology to systematically categorize the 128 skin conditions and facilitate expert evaluation in reader study3.

**Extended Data Fig. 9:**
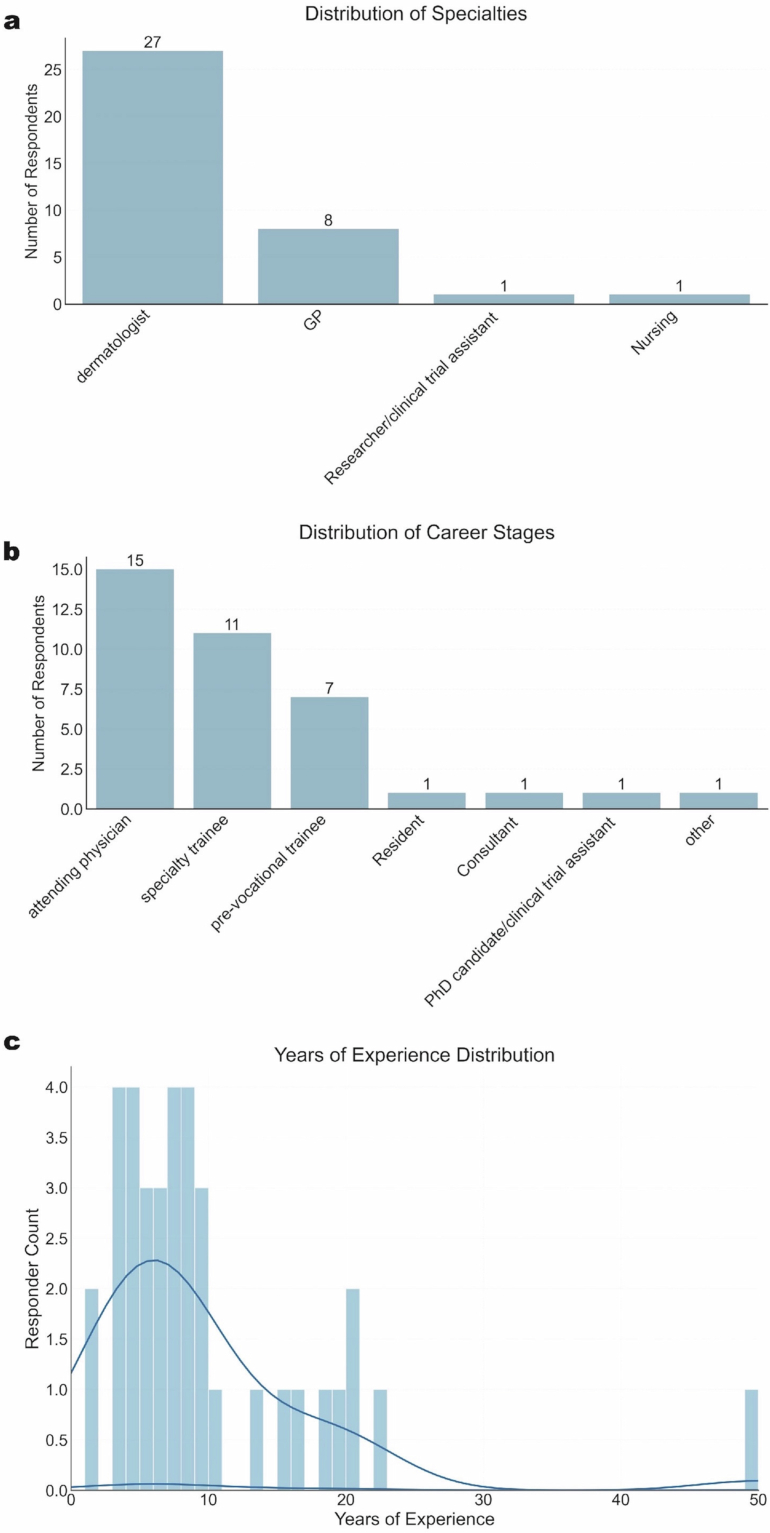
Demographic distribution of participants in reader study 3. **a**. Specialty distribution of participants. **b**. Career stage distribution of participants. **c**. Experience levels distribution by years.

We conducted a comprehensive reader study evaluating PanDerm’s diagnostic capabilities across 128 skin conditions using clinical photos. The study included 37 readers from 5 countries (Fig. 6b) and comprised 2 groups (Fig. 6a): the dermatology group (*n* = 20; 11 dermatology trainees and 9 specialists) and the generalist group (*n* = 17; 7 pre-vocational trainees, 8 GPs, 1 nurse and 1 clinical trial assistant). This grouping represents the distinction in specialty training backgrounds between dermatology-trained practitioners and those with general medical training. Each reader assessed up to 50 cases from a 200-case pool, providing their top 3 diagnoses both with and without PanDerm’s assistance. Four experienced dermatologists developed a standardized ontology for condition categorization (Extended Data Fig. 8). Performance was assessed primarily using 2 metrics: a 4-point diagnostic assessment scale for top 1 diagnosis (4, exact ontology match, to 1, significant mismatch) and top 3 diagnostic accuracy, with 3 independent dermatologists scoring and resolving discrepancies through panel review. PanDerm’s assistance significantly improved the average top 1 diagnostic scores of all readers from 2.83 to 3.08 (*P* *<* 0.001; Fig. 6c) and top 3 diagnostic accuracy from 54% to 63.4% (*P* *<* 0.001; Fig. 6d), while increasing readers’ diagnostic confidence (2.17 to 2.42, *P* *<* 0.001; Fig. 6e). The impact was particularly pronounced in the generalist group, showing higher diagnosis modification rates (28.6% versus 12.9% in the dermatology group; Fig. 6f) and greater improvements in both top 1 diagnostic scores (generalist group, +0.45; dermatology group, +0.25; Fig. 6g) and top 3 accuracy (generalist group, +16.5%; dermatology group, +10.3%; Fig. 6h). Analysis by condition classes showed consistent improvements across inflammatory, neoplastic and other categories (*P* *<* 0.05; Fig. 6i,j), with inflammatory conditions showing the largest gains (+0.36 in top 1 diagnostic scores, +14.2% in top 3 accuracy). Furthermore, when used independently, PanDerm achieved higher diagnostic accuracy than both unassisted readers (top 1 scores: 3.6 versus 2.83; *P* *<* 0.001) and human–AI collaboration (top 1 scores: 3.6 versus 3.08; *P* *<* 0.001). Further details on the setup, methodology, reader statistics and datasets of all three reader studies are provided in Methods, Extended Data Fig. 9 and Supplementary Tables 26–29.

## Discussion

Despite significant advances in AI technology, its application in clinical medicine remains fragmented and underutilized. Current AI systems are often restricted to isolated tasks and are unable to address the diverse demands of medical decision-making. This limits the potential of AI in supporting clinicians in disease diagnosis and management. Dermatology, with its complex requirements, including holistic patient assessment, lesion-specific analysis and potential use of various imaging modalities, serves as an ideal use case for showing AI’s capabilities across multiple interconnected clinical tasks. Success in this domain could pave the way for broader adoption of AI models across healthcare.

In this study, we introduce PanDerm, a versatile dermatology foundation model trained through self-supervised learning on over two million multimodal dermatological images. Central to PanDerm’s development was the curation of a large and diverse image dataset sourced primarily from in-house collections and carefully selected public repositories. This approach contrasts with previous efforts, such as SwAVDerm^35^, which relied on web-sourced skin data, inadvertently incorporating images from commonly used benchmarks such as ISIC^55^ and DermNet^44^, increasing the risk of data leakage and compromising evaluation validity. Our strategy minimizes this risk, ensuring that benchmark evaluations accurately reflect real-world model performance.

To evaluate PanDerm’s clinical utility, we conducted validations across 28 benchmark datasets, spanning comprehensive skin cancer assessment and a diverse set of primary care dermatological conditions. For skin cancer-related assessment, PanDerm outperformed existing models in specialized tasks across various modalities, including risk stratification of lesions, phenotype assessment, detection of lesion changes and malignancy, multi-class cancer diagnosis, lesion segmentation, and metastasis prediction and prognosis. In particular, PanDerm achieved the most results using only 10% of the task-specific training data typically required by existing models, helping alleviate the scarcity of specialist-labeled data in medical AI. In primary care dermatology settings, PanDerm also outperformed comparative models in diagnosing a diverse set of conditions such as inflammatory diseases, infectious conditions and frequently encountered dermatoses. These capabilities stem from its rich knowledge representation, developed through pretraining on varied dermatological image modalities and conditions, leading to consistent and significant performance improvements across tasks and modalities.

Three reader studies further supported these benchmark findings, suggesting PanDerm’s potential to assist clinical practice across different healthcare settings and specialty training backgrounds. In skin cancer diagnosis, PanDerm showed capabilities to improve diagnostic accuracy across clinicians of varying competence levels and identify concerning lesions before clinician detection—potentially facilitating earlier intervention. In general dermatology, PanDerm improved readers’ differential diagnosis across various skin conditions (for example, inflammatory dermatoses, cutaneous neoplasms and pigmentary disorders), with more substantial benefits observed among generalists (for example, primary care providers) evaluating inflammatory conditions—a considerable portion of everyday dermatological consultations. Given limited specialist access in primary care settings where most skin conditions are initially evaluated^56,57^, these findings indicate PanDerm’s potential to address dermatological expertise gaps across healthcare settings through both its technical capabilities and clinical applications. Importantly, across both human–AI collaboration studies, PanDerm alone performed equivalently to clinicians with PanDerm assistance in skin cancer diagnosis and even outperformed human–AI collaboration in differential diagnosis, similar to observations in a previous study^58^ showing ‘no significant difference between large language model (LLM)-augmented physicians and LLM alone’. This phenomenon probably stems from clinicians’ selective incorporation of AI recommendations rather than blind adherence, representing a balanced clinical implementation in which practitioners maintain their diagnostic autonomy while still benefiting from AI support.

The scaling behavior observed in PanDerm’s performance aligns with recent foundation model trends^20,22,23,59^, although achieving this in dermatology required addressing unique challenges in medical data acquisition and integration. Our analysis revealed two key technical insights: first, using CLIP^36^ as a teacher model achieved superior training data efficiency (Fig. 1f), outperforming the most representative method, DINOv2 (ref. ^38^)—particularly valuable given healthcare’s dataset limitations compared with the typical requirement of DINOv2 of 142 million images. Second, the masked feature reconstruction approach proved more effective at capturing subtle diagnostic features than methods such as MAE^19^. These advantages enabled PanDerm to improve upon both traditional models^42,60^ and recent generalist medical models such as BiomedGPT^41^. While generalist models advance broader biomedical AI, our results suggest that specialty-specific foundation models designed with clinical workflows in mind may offer more practical solutions for specialties in which multiple imaging modalities are crucial.

Despite promising results, we acknowledge several limitations in our evaluation scope and methodology. While our validation covered approximately 200 skin conditions across major categories (for example, inflammatory diseases, infections, neoplasms, benign growths, pigmented lesions and vascular anomalies), this represents only a fraction of known dermatological conditions (over 1,000 diagnoses) and is smaller than some previous studies (for example, ref. ^6^ with 445 conditions), with limited coverage of rare genetic disorders, complex systemic diseases and clinical variants. Regarding model robustness and fairness, while our benchmark evaluations (Supplementary Tables 30 and 31) show consistent performance across different settings (anatomical locations, age groups, genders and skin tones), several constraints exist: the evaluation mainly reflects overall accuracy rather than disease-specific analysis, has varying disease coverage across anatomical locations and focuses primarily on single imaging modalities. A more comprehensive evaluation framework^61^ integrating these aspects will be necessary for further assessing PanDerm’s robustness. Furthermore, recent studies^62–64^ have highlighted important challenges in dermatological AI systems, particularly in human–AI interactions. Although our evaluations show stable cross-skin-tone performance without explicitly balanced training data (as shown to be necessary in a previous study^63^), comprehensive bias assessment requires metrics beyond overall accuracy. Another study^64^ further revealed that equitable stand-alone performance may not translate to unbiased human–AI collaboration, which is crucial for clinical deployment. To address these limitations, future work should develop standardized protocols for cross-demographic evaluations using more comprehensive fairness metrics and investigate biases in human–AI collaborative settings. International collaborations such as ISIC^55^ will be crucial for creating representative datasets and establishing robust fairness standards.

In conclusion, PanDerm shows the potential of multimodal specialty-specific foundation models in addressing the diverse clinical needs across specialized and routine clinical practice in dermatology. Through comprehensive pretraining on diverse dermatological images and validation across multiple clinical scenarios, the model showed robust performance across different use cases. Our development approach, combining systematic data curation, advanced self-supervised learning and rigorous clinical validation, provides a framework for developing medical AI systems that can adapt to varying levels of clinical expertise and healthcare settings. These findings suggest promising directions for developing foundation models in other medical specialties in which the integration of diverse imaging modalities and complex clinical workflows is crucial for patient care.

## Methods

### Ethics statement

The MYM study was approved by the Metro South Health Human Research Ethics Committee on 21 April 2016 (approval number: HREC/16/QPAH/125). Ethics approval has also been obtained from the University of Queensland Human Research Ethics Committee (approval number: 2016000554), Queensland University of Technology Human Research Ethics Committee (approval number: 1600000515) and QIMR Berghofer (approval number: P2271). The HOP study has received approval from the Human Research Ethics Committee (HREC) from Metro South Health HREC (HREC/17/QPAH/816) and the University of Queensland HREC (2018000074). The ComBineMel dataset is part of the Computer Biomarkers Evaluation of Invasive Melanoma (ComBine Mel) study. The study was approved by the Alfred Hospital Ethics Committee on 8 August 2023 (approval number: HREC/98200/Alfred-2023). The study follows the National Statement on Ethical Conduct in Human Research (2007) protocols. The SDDI2 dataset has been approved by the Ethics Review Board of the Medical University of Vienna. The MMT data study is part of a research agreement study with Monash eResearch Centre and was approved through the Monash University Human Research Ethics Committee. The naevus surveillance study images (NSSI) dataset is part of the Brisbane Naevus Morphology Study, circa 2009–2014. The study followed the Declaration of Helsinki protocols and was approved by the Princess Alexandra Hospital human research ethics committee. The ACEMID pathology (ACEMID_path) pilot study has received approval from the Alfred Hospital Ethics Committee (approval number: 746/23) to share data accrued for registered trial ACTRN12619001706167 (ACEMID) under the Metro South Human Research Committee protocol HREC/2019/QMS/57206 and the University of Queensland Human Research Ethics Committee protocol 2019003077. The SDDI_Alfred study has received approval from the Alfred Hospital Ethics Committee (approval number: 198/19) for the use of sequential dermoscopic imaging data. Only de-identified retrospective data were used for research, without the active involvement of patients.

### Pretraining dataset for developing PanDerm

We curated an extensive pretraining dataset comprising 2,149,706 unlabeled multimodal skin images to develop PanDerm. This diverse dataset encompasses 4 imaging modalities and 11 data sources: 757,890 (35.3%) TBP tiles, 537,047 (25.4%) dermatopathology tiles, 460,328 (21.4%) clinical images and 384,441 (17.9%) dermoscopic images. This multimodality approach provides a comprehensive representation of skin lesions, enabling the model to learn robust features across different visual representations.

#### MYM cohort (TBP)

The MYM cohort^50^ is an in-house dataset studying the natural history of melanocytic nevi from 193 Australian participants recruited from the electoral roll. Three-dimensional (3D) TBP was conducted using VECTRA WB360 (Canfield Scientific), capturing 92 cross-polarized two-dimensional (2D) images with standardized lighting to create a 3D avatar. The average lesion tiles per subject was approximately 500. The final dataset comprises 405,856 automatically detected lesion image tiles ≥2 mm in diameter. Demographic information is available in Supplementary Table 32.

#### HOP cohort (TBP)

The HOP study^49^ is an in-house sequential dataset of high-risk melanoma individuals with 314 participants. Three-dimensional TBP imaging used the VECTRA WB360 system following the same protocol as MYM. Demographic and clinical data were collected through standardized questionnaires. More details about demographic information are available in Supplementary Table 33.

#### MYM and HOP cohort (dermoscopic)

These datasets also contain 38,110 dermoscopic images from suspicious lesions, providing complementary visualization of surface and subsurface structures potentially indicative of various skin conditions, particularly melanoma.

#### MMT dataset

The MMT dataset is an in-house collection amassed from over 150 clinics across Australia and New Zealand over a 15-year period. This extensive dataset primarily consists of paired polarized dermoscopic and clinical images. From this comprehensive collection, we curated a subset containing 316,399 dermoscopic images and 310,951 clinical images, providing a rich source of pretraining data for training purposes.

#### ACEMID pathology pilot study

This dataset comprises 54 patients from Queensland, Princess Alexandra Hospital (PAH) (48.1%) and New South Wales Melanoma Institute Australia (NSW MIA) (51.9%), with 57.4% males, aged 19–75 years (mean 53.4). Most patients (81.5%) were classified as ‘very high’ risk for melanoma, while others were ‘high’ risk (14.8%) or ‘low or average’ risk (1.9%). Lesions were predominantly nevi (68.5%, including common, dermal and congenital, and dysplastic, variants), melanomas (24.1%, mostly in situ) and other lesions (7.4%). While 66.7% had single lesions examined, others had 2–5 lesions per patient. Notable diagnostic variability between pathologists was observed. More details are available in Supplementary Table 34.

#### NSSI

NSSI is an in-house sequential collection of 29,832 dermoscopic images from 1,254 individuals in Brisbane, Australia (2009–2014). Images were collected using a digital dermatoscope attached to a Fotofinder ATBM imaging system (768 × 576 pixels at 96 dpi). The study included up to 7 time points per participant at 6-month intervals over 3 years. Individual lesions maintained consistent identification numbers across visits. See Supplementary Table 35.

#### Edu1 and Edu2

The Educational source 1 (Edu1) and Educational source 2 (Edu2) datasets comprise 81,947 and 67,430 clinical images, respectively, from in-house educational resources. They cover inflammatory and autoimmune disorders (psoriasis, atopic dermatitis), infections (herpes simplex, molluscum contagiosum, tinea corporis), pigmentary disorders (melasma, vitiligo), nail conditions (psoriatic nail disease, onychomycosis), vascular lesions (port-wine stains, pyogenic granulomas), and both benign and malignant tumors (melanoma, basal cell carcinoma, squamous cell carcinoma), including rare conditions and genetic disorders.

#### ISIC2024

ISIC2024 (ref. ^47^) is an open-source TBP-based dataset for identifying skin cancers among lesions cropped from 3D total-body photographs. We selected a subset containing 352,034 tile images, stratified by institutions.

#### TCGA-SKCM

The Cancer Genome Atlas—skin cutaneous melanoma (TCGA-SKCM) dataset^65^ from The Cancer Genome Atlas project characterized the mutational landscape of human skin cutaneous melanoma. It contains 475 slides processed into 377,764 patch images.

#### UAH89k

The UAH89k dataset^66^ includes 269 histopathology whole slide images from Heidelberg University, MVZ for Histology, Cytology and Molecular Diagnostics Trier, and the Institute for Dermatopathology, enriching the model’s understanding of skin conditions at the microscopic level.

### Detail of model architecture and pretraining

PanDerm is a self-supervised learning model designed for the dermatology field, built upon the success of existing self-supervised learning techniques in the natural image domain^67^. At its core, the architecture comprises a ViT-Large visual encoder^42^, a mask regressor and a CLIP-Large^36^ teacher model. The ViT-Large encoder, with its 24 transformer blocks and 1,024 dimensional embeddings, processes 224 × 224-pixel images, while the CLIP-Large teacher model handles slightly smaller 196 × 196-pixel inputs. The training process incorporates two primary objectives: masked latent alignment and visible latent alignment loss. Initially, the input image undergoes masking, with the mask ratio proportional to the encoder’s complexity (50% for ViT-Large). The encoder then processes visible patches to produce latent representations, while the regressor predicts the latent representations of masked patches using these visible latent and mask tokens. The model focuses on the encoder–regressor structure without a separate decoder component. The regressor assumes the responsibility of predicting the latent representations of masked patches, allowing for more efficient processing and learning. For target supervision, the unmasked image is fed through the CLIP model, generating supervision divided according to visible and masked patch locations. The visible latent alignment loss is directly applied to the latent representations of visible patches computed by the encoder. Concurrently, the masked latent alignment loss acts on the latent representations of masked patches predicted by the regressor. Both of these loss functions use CLIP latent representations as their supervision signals. The regressor in PanDerm operates similarly to a cross-attention mechanism. It uses learnable mask tokens as queries, while the keys and values are derived from the concatenation of visible patch representations and the output of previous layers. This design allows the regressor to effectively infer the content of masked regions based on the context provided by visible areas. Optimization primarily focuses on aligning the visible and masked patch predictions with their corresponding CLIP latent supervisions. This approach enables PanDerm to extract rich, semantically meaningful representations from dermatological images without relying on explicit labels.

For pretraining, we continued to train the model (initially trained on ImageNet-1K) on our dataset of over two million unlabeled multimodal skin images, representing diverse dermatological conditions. We set the batch size on each graphics processing unit (GPU) to 480, with an effective batch size of 1,920. Following masked image modeling practices^68^, we used a 50% mask ratio. To train our model, we used AdamW as the optimizer with an initial learning rate of 1.5 × 10^−3^. We apply simple data augmentation such as random resized cropping and horizontal flipping during pretraining. We trained our model for 500 epochs with a warmup of 20 epochs. The pretraining phase used 4 80-GB NVIDIA H100 GPUs and took approximately 5 days and 7 h. We chose the last epoch checkpoint as our final model weights. Please refer to Supplementary Table 36 for more detailed pretraining hyperparameter configurations.

#### Target representations (teacher model) of PanDerm

We tested different teacher models, including CLIP-base, CLIP-large, BiomedCLIP^40^ and MONET^39^ (dermatology-specific CLIP). CLIP-large outperformed biomedical-specific and dermatology-specific CLIP models, probably owing to the limited data scale of skin images in medical-domain CLIP models. Our model with CLIP-large teachers significantly improved performance and outperformed CLIP-large itself. See Supplementary Table 1 for detailed results.

#### Linear probing versus fine-tuning for PanDerm

We explored whether PanDerm’s features are ready for downstream tasks without fine-tuning, similar to DINOv2 (ref. ^38^) in the natural image domain. Our model using simple linear probing performed comparably with expensive full-parameter fine-tuning, suggesting that PanDerm’s features are already well suited for diverse downstream multimodal skin-related tasks without requiring further training. Detailed results are in Supplementary Table 2.

### Downstream evaluation details

#### Competing self-supervised learning baselines

For self-supervised learning methods comparison, we evaluated DINOv2 (ref. ^38^), MAE^19^ and MILAN^37^, all using the same ViT-Large backbone. We used the recommended hyperparameter configurations for these models and continued pretraining from their natural image training weights on our pretraining dataset. Subsequently, we fine-tuned these models using identical hyperparameter setups to ensure a fair comparison.

#### Fine-tuning and linear probing

In adapting PanDerm to downstream tasks, only the encoder model is used. For most tasks, PanDerm’s feature quality suffices to achieve competitive performance using simple linear probing. This involves applying a linear classifier (that is, logistic regression) to the top of extracted features from the PanDerm encoder to evaluate its performance on downstream tasks. For more challenging tasks requiring higher performance, we opted to fine-tune the PanDerm encoder. The fine-tuning tasks include the three reader studies, short-term change detection, skin lesion segmentation, skin cancer detection in ISIC2024 and TBP-based risk stratification. For all other tasks, we used linear probing. For linear probing, following practices recommended by the self-supervised learning community, we fix the *ℓ*_2_ regularization coefficient *λ* to *MC*/100, where *M* is the embedding dimension and *C* is the number of classes, and use the L-BFGS solver with a maximum of 1,000 iterations. For fine-tuning, we adhere to the BEiT V2 setting^68^, using cross-entropy loss with a learning rate of 5 × 10^−4^. We train models for 50 epochs with a warmup of 10 epochs. The model showing the best performance on the validation set is selected as the final model. For detailed hyperparameter configurations, please refer to Supplementary Table 37. In the following sections, we describe tasks with more specific methodological details.

#### Sequential data preprocessing for lesion change detection

Our proposed sequential data-preprocessing method consists of dark corner removal, skin inpainting, hair removal, image registration and lesion segmentation. For the first two steps, we follow the approach outlined in a previous study^69^. Given an image with or without dark corner artifacts, we convert it to grayscale and extract the contour using the OpenCV^70^ binary threshold function (threshold = 100) with the findContours function (RETR_TREE mode and CHAIN_APPROX_SIMPLE method). We identify the largest contour by calculating the area of all existing contours, capture a circular area using the minEnclosingCircle function, scale to 80% of the original radius and inpaint using the Telea algorithm (radius = 10). For hair removal, we convert to grayscale, apply a black hat morphological operation with a 17 × 17 structuring element, the threshold to create a binary mask, and inpaint. For image registration, we implement the AKAZE^71^ feature-based approach: detecting key points (descriptor size = 0, threshold = 9 × 10^−5^, octaves = 4), matching using the Brute Force matcher with Hamming distance, refining with RANSAC to estimate a EuclideanTransform model and warping using skimage.transform.warp with reflection padding and linear interpolation.

#### Siamese network for change detection

Similar to a previous study^45^, we use a simple Siamese network architecture for change detection, in which two identical visual encoders with shared weights from our foundation model process a pair of sequential lesion images captured over a short time frame. Each encoder extracts features from its respective image. These learned features are then concatenated and passed through two fully connected layers, followed by a softmax layer for final classification. For training this Siamese network in our binary change detection task, we use a contrastive loss function. This loss is particularly well suited for Siamese networks as it helps the model learn to distinguish between pairs of images that have changed and those that have not. The contrastive loss encourages the network to minimize the distance between feature representations of image pairs with no significant changes while maximizing the distance for pairs that show meaningful changes. This approach allows the network to learn a similarity metric between image pairs, rather than simply classifying individual images.

#### Melanoma metastasis prediction and survival analysis

We use a linear probing classifier on our foundation model to predict melanoma metastasis using dermoscopic images from the private ComBineMel dataset. Our evaluation encompasses two scenarios: binary metastasis prediction and multi-class metastasis prediction. In the binary classification, we aim to differentiate between the presence of any metastasis (including local, satellite and in-transit metastases, lymph node recurrence, and distant metastasis) and its absence. The multi-class prediction presents a more complex challenge, categorizing cases into three groups: control (no metastasis); local, satellite and in-transit metastases; and distant metastasis. To enhance the robustness and mitigate potential data selection bias, we perform five iterations of dataset splitting into training and testing sets, stratified by melanoma stage. The model is trained using these fivefold data. We linear probed PanDerm with the setting mentioned above. We then generated out-of-fold predictions for all lesions and compare these with the ground truth for performance evaluation.

Subsequently, we conduct a multivariate Cox regression analysis, incorporating the metastasis prediction score and clinical variables (age, sex, Breslow thickness, ulceration, dermal mitosis, melanoma subtype and lesion location) to predict the RFI. This analysis focuses on earlier stages of melanoma (stages I–II). We visualize the relative contribution of individual variables to prognosis prediction using a forest plot. To analyze the correlation between variables and RFI, we use the Kaplan–Meier method. Patients are stratified into low-risk and high-risk groups based on their binary metastasis prediction scores (median value). The log-rank test is used to assess the classifier’s ability to predict survival. To evaluate the predictive accuracy at various time points, we generate time-dependent receiver operating characteristic curves and calculate AUCs at 3 years, 5 years and 7 years.

#### Melanoma screening using TBP

The melanoma screening algorithm is designed to identify high-risk lesions among whole-body images, aiding clinicians in efficiently detecting potential malignancies. Lesions flagged as high risk undergo further triage and dermoscopic examination. The screening model integrates three modules: a risk prediction head, a UD detection head and a machine learning module, using both TBP image data (image tiles) and metadata for comprehensive predictions. We first fine-tune our foundation model, equipped with the risk prediction head, using TBP image tiles to classify lesions as high risk or low risk. All lesion images are resized to 224 × 224 pixels and subjected to data augmentation, including color and geometric transformations. The risk prediction head, comprising a single linear layer, identifies lesions as high risk if subjected to dermoscopy examination and low risk otherwise. The UD detection head leverages the ‘UD sign’, an effective diagnostic strategy that compares all lesions from the same patient to identify outliers. This approach capitalizes on lesion contextual information. We use the fine-tuned foundation model to extract deep learning features, which are then processed by the UD detection head. This module calculates the distance between each lesion’s features and the average features of all lesions from the same patient, using the interquartile range method to select outlier lesions. The machine learning module, an extra tree classifier, is trained using TBP metadata, which include 32 measurements for each lesion from the 3D TPB machine. This module directly predicts malignancy based on pathology labels. The final screening result combines predictions from all three modules. A lesion is flagged as suggestive of malignancy if any module yields a positive prediction. We evaluate the screening performance at both the lesion and patient levels to ensure comprehensive accuracy assessment.

#### Weakly supervised slide classification

Weakly supervised slide classification tasks are approached using the established two-stage multiple instance learning framework: (1) extracting instance-level features from tissue regions within the whole slide image (WSI) and (2) developing an order-invariant aggregation method to consolidate patch-level data into slide-level representation. For preprocessing, we use the CLAM toolbox^72^ for tissue segmentation, partitioning regions into 256 × 256 nonoverlapping sections at ×20 magnification, then resizing to 224 × 224 and normalizing using ImageNet parameters. To evaluate pretrained encoders, we implement the attention-based multiple instance learning algorithm^73^ with consistent configurations. Our implementation features a two-tier gated ABMIL structure with an initial FC layer mapping to 512-dimensional space, followed by intermediate layers with 384 hidden units. We incorporate dropout regularization (rates 0.10 and 0.25), use the AdamW optimizer^74^ with a cosine learning rate schedule (initial rate 1 × 10^−4^, weight decay 1 × 10^−^^5^), and use cross-entropy loss. Training runs for 20 epochs with early stopping based on validation loss. We ensure robust evaluation through fivefold cross-validation, stratifying by both case and label attributes.

#### Skin lesion segmentation

For skin lesion segmentation, we use a conventional segmentation paradigm, using a network encoder connected to a segmentation decoder and head. Our proposed PanDerm serves as the encoder in this setup. We benchmark PanDerm against three established models: ViT-Large^42^, autoSMIM^33^ and BATFormer^75^. Both ViT and PanDerm use an UperNet decoder, following the official ViT implementation. For autoSMIM and BATFormer, we adhere to their official repository settings. ViT-Large and autoSMIM encoders are initialized with ImageNet pretrained weights. To ensure a fair comparison, all images are resized to 224 × 224. We apply online data augmentation, including color jittering, random rotation and random flipping, to mitigate overfitting. The training uses an AdamW optimizer with an initial learning rate of 5 × 10^−4^ and a weight decay of 0.01, with the learning rate decaying according to a cosine schedule. The models are trained for 100 epochs, and we save the model that achieves the best evaluation metrics on the validation set.

#### Early melanoma detection (reader study 1)

We fine-tuned our foundation model on the private SDDI–Alfred dataset^54^ using a tenfold cross-validation approach. We used cross-entropy loss with a learning rate of 5 × 10^−4^. We train models for 50 epochs with a warmup of 10 epochs. The model showing the best AUROC on the validation set is selected as the final model. We then used an out-of-fold prediction approach to generate melanoma predictions for all sequential images. For each image sequence, we recorded the time point at which the model first made a correct diagnosis of melanoma; otherwise, the model was considered to have failed in detecting the melanoma. While biopsy serves as our reference standard, we aimed to explore the algorithm’s potential to detect early signs of melanoma progression. Our study focused on identifying suspicious changes in sequential images before clinical diagnosis, with the goal of enabling earlier intervention when melanomas are most treatable. For the human evaluation, 12 clinicians—seven dermatologists with over 5 years of experience and five dermatology residents with less than 5 years of experience—were invited to assess the serial dermoscopic data. The images were presented to the reviewers using Qualtrics (Provo), with the reviewers blinded to the true diagnoses. For each case, information such as the patient’s age, sex, lesion location and date of imaging was provided. Initially, only the first dermoscopic image in the sequence was shown, and reviewers were asked to classify the lesion as either benign or malignant. As they progressed through the sequence, side-by-side image comparisons were made available to assess changes over time. Once a diagnosis was submitted, it could not be revised. To mitigate bias, we included ten single time-point melanoma images, preventing reviewers from assuming that the first image in a series was benign. We then compared the diagnostic performance of the clinicians with our model, focusing on the time point at which a malignant diagnosis was first made by either the clinicians or the algorithm.

#### Human–AI collaboration for skin cancer diagnosis

The reader study was conducted using DermaChallenge, a web-based platform developed and hosted by the Medical University of Vienna for online education on dermatoscopy, as described in previous studies^76,77^. To ensure proper authentication and data management, readers were required to register with a unique username, valid email address and password. Active users on the platform, who previously actively agreed to be contacted, were recruited via a single email. Before commencing the study phase, all users had to finish three introduction levels to be familiarized with the platforms’ user interface and image types. The number of correct answers in the first iteration of these levels, normalized against the mean score of the entire DermaChallenge platform user base, served as a score of experience. Users were grouped into ‘low’ (*n* = 11), ‘medium’ (*n* = 21) and ‘high’ (*n* = 9) experience based on quantiles with cuts at 0.25 and 0.75 probability (R stats::quantile() function). Within the study level, users were shown batches of 10 images, randomly selected from a pool of 1,511 images, that is, the ISIC 2018 Task 3 test set, with a predefined diagnosis distribution (actinic keratosis and intraepidermal carcinoma (AKIEC): 1, basal cell cacinoma (BCC): 1, benign keratinocytic lesion (BKL): 1, dermatofibroma (DF): 1, vascular lesion (VASC): 1, melanoma (MEL): 2, melanocytic nevus (NV): 3). For each image, a user had to choose one diagnosis out of seven options, and subsequently again after assistance from our foundation model, presented as multi-class probabilities visualized as bars and numbers for each class. Readers had the flexibility to complete multiple survey rounds with different image batches at their discretion; incompletely answered batches were omitted. The study was conducted online from 20 August to 12 September 2024, during which we collected data from 41 raters. Our foundation model for decision support used a weighted random sampler strategy, following the approach from^76^ but excluding test-time augmentation. The model showed robust performance, achieving an 80.4% mean (macro-averaged) recall, with notably high recall rates for critical skin lesions: 87.2% for melanoma and 86.0% for BCC.

#### Human–AI collaboration for 128 skin condition diagnoses

The reader study was conducted using a web-based platform developed for online dermatological assessment. A total of 37 healthcare professionals participated in the study, categorized into two groups based on specialization: a dermatology group (*n* = 20) comprising 9 dermatology specialists and 11 specialty trainees, and a generalist group (*n* = 17) including 7 GPs, 7 general medicine practitioners and 3 other healthcare professionals (nursing, clinical trial assistants) who manage skin conditions within their broader practice scope. This grouping strategy reflects the real-world clinical setting in which nondermatologist healthcare professionals routinely perform initial skin assessments. The diverse range of 128 skin conditions enabled the evaluation of diagnostic performance between dermatologically trained professionals and those with general medical training. Readers were presented with clinical images and asked to provide their assessment through a structured questionnaire. Each participant rated image quality on a 5-point scale (from ‘not at all’ to ‘completely’ assessable), provided a primary diagnosis through free-text entry and optionally listed two differential diagnoses ranked by likelihood. Diagnostic confidence was recorded on a 4-point scale (1, not at all confident; 2, somewhat confident; 3, confident; 4, highly confident). Following their initial assessment, readers were shown PanDerm’s top 3 predicted diagnoses and given the opportunity to maintain or modify their original diagnosis and differential diagnoses, followed by a reassessment of their confidence using the same 4-point scale. The study collected 1,342 responses between 1 July and 2 October 2025. Before the evaluation, four experienced dermatologists collaboratively developed a standard ontology to systematically categorize the 128 skin conditions and facilitate expert evaluation (Extended Data Fig. 8). The evaluation process involved multiple expert assessors who independently scored diagnostic accuracy using a 4-point scale: 4, direct match with the predefined term in the ontology; 3, match within the same diagnostic category in the ontology; 2, inconsequential misdiagnosis; and 1, significant mismatch, potentially dangerous misdiagnosis. To ensure robust assessment, each case was evaluated by three assessors, with cases showing significant scoring discordance (differences between 3/4 and 1/2) reviewed in consensus meetings to establish final scores. For the top 3 accuracy evaluation, both human readers and AI assistance were evaluated based on whether the correct diagnosis appeared within their top 3 diagnostic choices.

#### Evaluation metrics

For multi-class tasks, we primarily use a weighted F1 score, which averages class-specific F1 scores (harmonic means of precision and recall) weighted by class size. It addresses class imbalance in multi-class scenarios. For binary classification, we primarily use AUROC, measuring the model’s ability to distinguish between classes across all classification thresholds. An AUROC of 1.0 indicates perfect classification, while 0.5 suggests random guessing. This metric is particularly useful for imbalanced datasets and when we need to evaluate trade-offs between true-positive and false-positive rates. For the three reader studies, we report accuracy (top 1 or top 3). In skin lesion segmentation, we use the Dice similarity coefficient and Jaccard index to assess segmentation quality. For TBP-based melanoma screening, we primarily report the sensitivity (recall) in malignant lesions, focusing on the model’s ability to correctly identify malignant cases.

#### Statistical analysis

For skin tumor patch classification, melanoma slide classification, reader studies, metastasis prediction and skin lesion segmentation, we conduct *k*-fold cross-validation owing to either a relatively small sample size or following conventional practice. We compute the mean and standard deviation of performance across the folds, then calculate the standard error by dividing the standard deviation by the square root of the number of folds. The 95% CI is derived using 1.96 times the standard error. To assess statistical significance, we conduct two-sided *t*-tests comparing PanDerm’s performance against the baseline model for each task. For the remaining datasets, we use nonparametric bootstrapping with 1,000 replicates to estimate 95% CIs for each model’s performance. To compare models, we implement pairwise permutation tests, conducting 1,000 permutations per pair and recalculating performance metrics after each permutation. We derive two-sided *P* values to evaluate the null hypothesis that paired observations stem from identical distributions. In addition, we perform *t*-tests to assess the statistical significance of inter-model performance variations. Our null hypothesis posits no discernible difference between PanDerm’s performance and that of its competitors. *P* < 0.05 was regarded as statistically significant.

### Skin cancer and general skin condition classification datasets

#### HAM10000 (7 classes)

The HAM10000 (ref. ^34^) dataset contains 10,015 dermoscopic images across 7 classes: actinic keratoses, basal cell carcinoma, benign keratosis, dermatofibroma, melanocytic nevi, melanoma and vascular lesions. It is stratified into 60% training, 20% validation and 20% test sets. For human–AI collaboration, we used the official dataset. All other experiments used the clean version from a previous study^78^, which prevents data leakage by ensuring that lesions from the same patient are not split across sets.

#### BCN20000 (9 classes)

The BCN20000 (ref. ^79^) dataset comprises 12,413 dermoscopic images in 9 categories: nevus, melanoma, basal cell carcinoma, seborrheic keratosis, actinic keratosis, solar lentigo, squamous cell carcinoma, dermatofibroma and vascular lesions, including lesions in hard-to-diagnose locations. It is similarly stratified (60–20–20 split). We used the clean version of BCN20000, which, like the HAM10000, addresses data leakage issues.

#### MSKCC (2 classes)

The Memorial Sloan Kettering Cancer Center (MSKCC)^55^ dataset is curated from the MSKCC data from the ISIC archive^55^, containing 8,984 dermoscopic images with melanoma and other classes.

#### HIBA (2 classes)

The HIBA^55^ dataset is curated from the HIBA data from the ISIC archive^55^, containing 1,635 dermoscopic images with melanoma and other classes.

#### PAD-UFES-20 (6 classes)

The PAD-UFES-20 (ref. ^43^) dataset from Brazil contains 2,298 close-up clinical images with 6 classes, including actinic keratosis, basal cell carcinoma of the skin, malignant melanoma, melanocytic nevus of the skin, squamous cell carcinoma and seborrheic keratosis.

#### DDI (2 classes)

We grouped the classes of the diverse dermatology images (DDI) dataset^63^ into melanoma and others. The dataset contains 647 clinical images from the United States.

#### Derm7pt (2 classes)

Derm_D is a subset of Derm7pt (ref. ^80^), containing 839 dermoscopic images, and Derm_C contains 839 clinical images with melanoma and other classes.

#### ISIC2024 (2 classes)

ISIC2024 (ref. ^47^) is a multicenter dataset with skin lesion crops from TBP. We chose holdout data with 49,025 crop images with three institutions (FNQH Cairns, Alfred Hospital, Melanoma Institute Australia) as the evaluation dataset.

#### PH2 (3 classes)

PH2 (ref. ^81^) is a clinical image dataset from Portugal with 200 images and 3 classes. We reorganize it to a binary melanoma detection task.

#### Med-Node (2 classes)

The Med-Node^82^ dataset contains 170 clinical images. We reorganize it to a binary melanoma detection task.

#### DermNet (23 classes)

DermNet^44^ contains 19,559 clinical images; this dataset consists of images of 23 types of skin diseases and captures common clinical presentations including inflammatory conditions (eczema, psoriasis), infections (bacterial, viral, fungal) and neoplastic diseases.

#### Fitzpatrick17K (114 classes)

The Fitzpatrick17K (ref. ^62^) dataset comprises 16,577 clinical images annotated with both dermatological diagnoses and Fitzpatrick skin types (I–VI). It encompasses 114 distinct conditions (minimum of 53 images per condition) spanning major dermatological categories: inflammatory dermatoses (psoriasis, lichen planus, various eczematous conditions), cutaneous malignancies (melanoma, morpheiform and solid-cystic variants of BCC, SCC), papulosquamous disorders (pityriasis rosea, pityriasis rubra pilaris), autoimmune conditions (lupus erythematosus, bullous diseases), benign neoplasms (seborrheic keratosis, dermatofibroma) and various other clinically significant entities (acanthosis nigricans, granuloma annulare, necrobiosis lipoidica).

#### MMT-09 (9 classes)

The dataset is an in-house clinical dataset with 9 skin condition classes, including benign keratinocytic, malignant keratinocytic, melanocytic, inflammatory conditions and benign tumors, vascular lesion, basal cell carcinoma, malignant keratinocytic, melanoma and squamous cell carcinoma. We chose 38,476 images as our evaluation dataset.

#### MMT-74 (74 classes)

The MMT-74 dataset (Supplementary Table 38) is a comprehensive in-house clinical collection comprising 38,476 dermatological images across 74 detailed skin condition classes, building upon and refining the broader 9-class structure of MMT-09. This structured dataset encompasses diverse dermatological conditions, including detailed classifications of basal cell carcinoma variants (nodular, pigmented, superficial and recurrent), melanocytic lesions with specific pattern recognition (such as acral patterns and various nevus types), inflammatory disorders (dermatitis, psoriasis), benign proliferations (including seborrheic keratosis variants) and vascular lesions (angiomas, telangiectasias). The dataset was specifically designed to evaluate deep learning models’ performance across a diverse and clinically relevant range of skin conditions, with categories spanning inflammatory, infective, benign proliferations, melanocytic and eczema classifications.

#### SD-128 (128 classes)

This dataset encompasses 5,619 clinical images covering 128 dermatological conditions spanning the complete spectrum of clinical practice. The dataset provides substantial coverage of inflammatory dermatoses, ranging from common presentations (such as psoriasis and atopic dermatitis) to less common entities (such as leukocytoclastic vasculitis). It includes diverse infectious diseases of bacterial, viral and fungal etiologies, as well as a comprehensive range of proliferative lesions from benign nevi to malignant melanomas. The collection also extends to appendageal disorders, physical-trauma-related changes, nail disorders and hair-loss conditions. This extensive compilation represents both frequently encountered conditions in everyday practice and challenging rare cases, providing a robust resource for clinical diagnostic support. This dataset contains 5,619 clinical images encompassing diverse dermatological conditions commonly encountered in clinical practice. The dataset provides substantial coverage of inflammatory conditions from common presentations (psoriasis, atopic dermatitis) to less common entities (leukocytoclastic vasculitis); various infectious diseases spanning bacterial, viral and fungal etiologies; and a range of proliferative lesions from benign nevi to malignant melanomas as well as appendageal disorders and physical-trauma-related changes. We used 10% of the data stratified by disease labels for benchmark evaluation. In addition, we selected 200 images stratified by disease classes for our reader study.

#### Skin tumor patch classification (PATCH16) (16 classes)

The skin tumor patch classification task^66^ consists of tissue patches of 378 histopathology WSIs from the archive of the Institute of Pathology, Heidelberg University, the MVZ for Histology, Cytology and Molecular Diagnostics Trier and the Institute for Dermatopathology Hannover for classification of 16 categories including 4 tumor types and 12 normal tissue structures. We obtained a total of 129,364 image patches of 100 × 100 μm (395 × 395) size. The dataset was stratified by label, with 55% allocated for training, 15% for validation and 30% for testing.

#### Melanoma slide classification (WSI) (2 classes)

The melanoma slide classification task^83^ from the National Cancer Institute’s Clinical Proteomic Tumor Analysis Consortium Cutaneous Melanoma (CPTAC-CM) cohort consists of histopathology WSIs for cancer detection. After selecting labeled WSIs, we obtained 302 slides (71 normal, 231 tumor). For training and evaluation, we used a fivefold cross-validation strategy with label-stratified splits to maintain class balance.

#### Early melanoma detection based on SDDI–Alfred (2 classes)

The dataset (Supplementary Table 39) consists of 179 serial dermoscopic imaging sequences from 122 patients, totaling 730 dermoscopic images. The patients were recruited from a private specialist dermatology clinic, with follow-up periods ranging from January 2007 to December 2019. The study population showed distinct characteristics between melanoma and benign groups: patients with melanoma had a mean age of 56.6 years (s.d. = 11.8) compared with 49.6 years (s.d. = 11.4) in the benign group, with slightly different gender distributions (53.9% male in melanoma versus 40.0% male in benign cases). Both melanoma and benign lesions that underwent short- or long-term SDDI at least once before biopsy were included. The dataset is well balanced, with 90 benign lesions and 89 malignant lesions. Of the 89 melanomas, 34 (38.2%) were invasive, with a mean Breslow thickness of 0.5 mm, while 55 (61.8%) were in situ. The melanoma subtypes included invasive superficial spreading melanoma (SSM) (36.0%), in situ SSM (31.4%), unspecified in situ (18.0%), lentigo maligna (12.3%) and invasive lentigo maligna melanoma (LMM) (2.2%). The benign lesions were predominantly dysplastic nevi (40.0%), followed by compound nevi (27.8%), junctional nevi (18.9%) and intradermal nevi (8.9%). Anatomically, lesions were most commonly located on the lower limb (29.2% melanoma, 26.7% benign) and back (23.5% melanoma, 25.6% benign). All lesions were monitored via digital dermoscopy, excised owing to clinical concerns and confirmed by pathological examination. The number of images per sequence varied from 1 to 12, with an average of approximately 4 images per sequence.

### Longitudinal and melanoma metastasis datasets

#### Short-term lesion change detection based on SDDI1 (2 classes)

The SDDI1 (ref. ^55^) dataset is sourced from the ‘Repeated Dermoscopic Images of Melanocytic Lesions’ by University Hospital Basel, available in the ISIC archive. It comprises 116 sequential lesions, each with a sequence length of 5, from 66 patients. The dataset is categorized into two classes for lesion change detection.

#### Lesion change detection based on SDDI2 (2 classes)

SDDI2 is an in-house dataset from the Medical University of Vienna. It contains 229 sequential dermoscopic images with a sequence length of 2. The dataset includes both binary change labels and more fine-grained malignant change labels. This dataset is also used for short-term lesion-change detection.

#### Melanoma metastasis and survival prediction (2 or 3 classes)

The ComBineMel dataset encompasses 680 dermoscopic images of invasive melanoma from 370 patients recruited across 10 hospital sites in multiple countries, including Australia and 5 European nations. For large melanomas, multiple images were captured to ensure comprehensive coverage of the entire lesion area. The study population is included in Supplementary Table 40. Regarding disease staging, the majority of cases were classified as stage I (70.5%), followed by stage III (16.5%), stage II (12.2%) and stage IV (0.8%). In terms of T classification, T1a was the most common (59.2%), followed by T2a (18.6%) and T4b (13.2%). Sentinel lymph node biopsy was not performed in most cases (71.6%), with 10.8% positive and 17.6% negative results among those tested. For nodal status, N1 disease was the most common (10.8%), followed by N2 (3.8%) and N3 (1.8%). Regarding metastasis status, 248 (67.0%) of cases showed no metastasis, while 66 (17.8%) presented with metastasis at the time of diagnosis. In addition, 56 (15.1%) of cases developed metastasis during the follow-up period.

#### Skin lesion segmentation based on ISIC2018 and HAM10000

The skin lesion segmentation task is evaluated using two publicly available datasets. The ISIC2018 dataset^52^ comprises 3,694 dermoscopic images with 2,594 images for training, 100 for validation and 1,000 for testing. We follow this official dataset split for our experiments. The HAM10000 dataset^34^ includes 10,015 dermoscopic images, each with corresponding binary segmentation labels. A randomized selection approach is adopted, with 64% of the images used for training, 16% for validation and the remaining 20% for testing.

### 3D TBP datasets

This dataset comprises 3D TBP images captured using the VECTRA WB360 system (Canfield Scientific). The system uses 92 cameras to simultaneously capture cross-polarized 2D images with standardized lighting within seconds, which are then merged to create a high-fidelity 3D avatar of each patient’s entire skin surface. From these 3D avatars, individual lesion tiles were exported for further analysis. Unlike stand-alone clinical photographs, TBP represents a higher-order imaging modality in which 2D tiles are systematically derived from 3D reconstructions, maintaining spatial relationships. The standardized acquisition with calibrated lighting enables the capture of the entire body surface with overlapping views, providing consistent anatomical landmarks and contextual information for comprehensive assessment, including skin phenotype patterns, lesion measurements and ‘UD’ sign application. The images undergo calibration and stitching, resulting in standardized 2D tiles with consistent quality across all body regions.

#### Photodamage risk assessment datasets (3 classes)

This in-house dataset^84^ contains image tiles (693 × 693 pixels) created from 92 raw 2D photos, each representing approximately 10 cm^2^ of cutaneous surface. Tiles with <33% skin surface were excluded using pixel color analysis. Manual review removed out-of-focus images, tiles with multiple body sites or identifying features. The final dataset comprises 5,022 image tiles from MYM^50^ and HOP^49^ studies, labeled as low, moderate or severe photodamage risk labeled primarily by dermatology students.

#### Nevus counting datasets (2 classes)

This dataset, derived from the in-house MYM^50^ study, contains 28,227 lesion tiles annotated as nevus or nonnevus. Three expert physicians independently labeled lesions on-screen, with consensus determined by ≥2 clinicians’ agreement. A senior dermatologist manually identified nevi in-clinic using a dermatoscope, serving as the gold standard for the test set. To ensure consistency, lesions under underwear, on the scalp or on foot soles were excluded, and only lesions ≥2 mm were considered. A minimum 1-month interval was maintained between on-screen and in-clinic labeling sessions.

#### Risk prediction and TBP screening datasets (2 classes)

This dataset comprises 2,038 TBP scans from 480 patients, collected from the MYM and HOP studies. The raw TBP scans include nevi images and a variety of nonrelevant images such as normal skin, scars and freckles. To focus only on nevi, we applied filtering parameters based on built-in Vectra data settings: majorAxisMM ≥ 2, deltaLBnorm ≥ 4.5, out_of_bounds_fraction ≤ 0.25, dnn_lesion_confidence ≥ 50 and nevi_confidence > 80. This process resulted in 196,933 lesion image tiles. We stratified the data by the patient for training, validation and testing: 360 patients for training (146,752 images), 40 patients for validation (19,483 images) and 80 patients for testing (30,698 images, including 28 malignant lesions). Of the total dataset, 216 images represent malignant lesions, with 40 confirmed melanoma cases.

#### Measurements in TBP

Alongside the image tiles, Vectra provides a range of measurements for each lesion, mainly including size, color and shape. Our TBP screening model incorporates 32 such measurements: ‘A’, ‘Aext’, ‘B’, ‘Bext’, ‘C’, ‘Cext’, ‘H’, ‘Hext’, ‘L’, ‘Lext’, ‘areaMM2’, ‘area_perim_ratio’, ‘color_std_mean’, ‘deltaA’, ‘deltaB’, ‘deltaL’, ‘deltaLB’, ‘deltaLBnorm’, ‘dnn_lesion_confidence’, ‘eccentricity’, ‘location_simple’, ‘majorAxisMM’, ‘minorAxisMM’, ‘nevi_confidence’, ‘norm_border’, ‘norm_color’, ‘perimeterMM’, ‘radial_color_std_max’, ‘stdL’, ‘stdLExt’, ‘symm_2axis’ and ‘symm_2axis_angle’.

### Computing hardware and software

Scripts for data collection and processing were written in Python (version 3.9.19) using the libraries Pandas (version 2.2.2), Numpy (version 1.26.4) and Pillow (version 10.3.0). For self-supervised pretraining, we used 4 × 80 GB NVIDIA H100 GPUs configured for multi-GPU single-node training using DistributedDataParallel (DDP) as implemented by Python (v.3.9.13), PyTorch (v.2.2.1, CUDA 11.8) and Torchvision (v.0.17.1). The CAE-v2 code is used as the codebase to develop our foundation model, which can be found in its official repository (https://github.com/Atten4Vis/CAE). For downstream task evaluation, all experiments were conducted on 4 × 49 GB NVIDIA 6000 Ada GPUs. We used Python (v.3.9.19), PyTorch (v.2.2.2, CUDA 11.8) and Torchvision (v.0.17.2) for fine-tuning tasks, and Python (v.3.10.14), PyTorch (v.2.2.2, CUDA 11.8) and Torchvision (v.0.17.2) for linear probing tasks. We used Scikit-learn (v1.2.1) for logistic regression in the linear probing setting. Implementation of other comparative pretrained models was modified based on the official configuration in their respective repositories: MAE (https://github.com/facebookresearch/mae), SL_ImageNet (https://huggingface.co/timm/vit_large_patch16_224.orig_in21k), DINOv2 (https://github.com/facebookresearch/dinov2), SwAVDerm (https://github.com/shenyue-98/SwAVDerm), autoSMIM (https://github.com/Wzhjerry/autoSMIM), BATFormer (https://github.com/xianlin7/BATFormer), MedSAM (https://github.com/bowang-lab/MedSAM), ResNet50 (https://pytorch.org/vision/main/models/generated/torchvision.models.resnet50.html), MILAN (https://github.com/zejiangh/MILAN), CLIP (https://github.com/openai/CLIP), BiomedCLIP (https://huggingface.co/microsoft/BiomedCLIP-PubMedBERT_256-vit_base_patch16_224) and MONET (https://github.com/suinleelab/MONET/tree/main).

### Reporting summary

Further information on research design is available in the Nature Portfolio Reporting Summary linked to this article.

## Online content

Any methods, additional references, Nature Portfolio reporting summaries, source data, extended data, supplementary information, acknowledgements, peer review information; details of author contributions and competing interests; and statements of data and code availability are available at 10.1038/s41591-025-03747-y.

## Supplementary information

Supplementary Information Supplementary Tables 1–40.

Reporting Summary

## Figures and Tables

**Extended Data Table 1:**
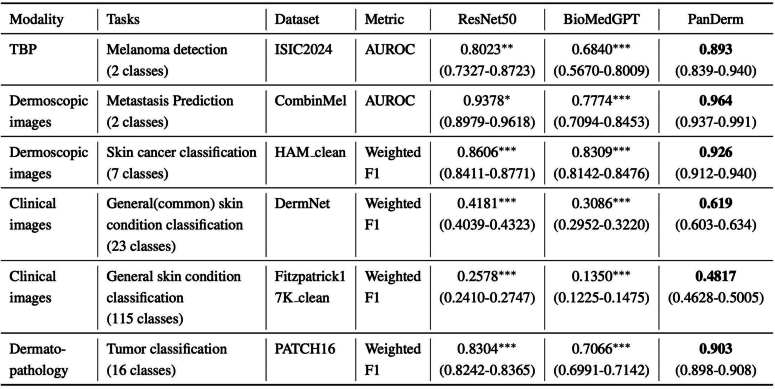
Performance comparison between PanDerm and BiomedGPT across different dermatological tasks and modalities

Models are evaluated on various dermatological tasks spanning TBP, dermoscopic images, clinical photographs, and dermatopathology. Evaluation metrics include Area Under the Receiver Operating Characteristic curve (AUROC) for binary classification tasks and Weighted F1 score for multi-class classification. Performance is reported with 95% confidence intervals in parentheses. The best performance for each task is bolded. *** p < 0.001 compared to PanDerm. P-values calculated using a two-sided t-test.

## Data Availability

Most datasets used in this study are publicly available. These datasets used for skin lesion diagnosis and segmentation tasks can be accessed through various repositories. The ISIC archive (https://www.isic-archive.com/) hosts several datasets, including MSKCC and HIBA. Other widely used benchmark datasets are available through their respective portals: BCN20000 (https://figshare.com/articles/journal_contribution/BCN20000_Dermoscopic_Lesions_in_the_Wild/24140028/1), PAD-UFES-20 (https://www.kaggle.com/datasets/mahdavi1202/skin-cancer), DDI (https://ddi-dataset.github.io/index.html), Derm7pt (https://derm.cs.sfu.ca/Welcome.html), ISIC2024 (https://www.kaggle.com/competitions/isic-2024-challenge), Med-Node (https://www.kaggle.com/datasets/prabhavsanga/med-node), DermNet (https://www.kaggle.com/datasets/shubhamgoel27/dermnet), WSI (https://portal.gdc.cancer.gov/projects/TCGA-SKCM), PATCH16 (https://heidata.uni-heidelberg.de/dataset.xhtml?persistentId=doi:10.11588/data/7QCR8S), ISIC2018_task1 and HAM10000 (https://challenge.isic-archive.com/data/), SDDI1 (https://api.isic-archive.com/collections/328/), PH2 (https://www.fc.up.pt/addi/ph2%20database.html), SD-128 (https://huggingface.co/datasets/resyhgerwshshgdfghsdfgh/SD-198) and UAH89k (https://heidata.uni-heidelberg.de/404.xhtml;jsessionid=6a9c0981ef8e0874c5dca6e1600a). Access to in-house datasets is restricted due to patient privacy considerations. These include MMT for dermoscopic and clinical image pretraining and downstream multi-skin condition classification, NSSI for sequential dermoscopic image pretraining, ACEMID_path for dermatopathology pretraining, Edu1 and Edu2 for clinical image pretraining, SDDI2 for lesion change detection, SDDI_Alfred for reader study 1 (early melanoma detection) and the TBP data from MYM and HOP studies for all TBP-based pretraining and evaluation. Researchers interested in accessing these datasets should direct their requests to the corresponding author. All requests will receive a response within 2 weeks of submission. Requests will be evaluated according to institutional and departmental policies to ensure compliance with intellectual property rights and patient privacy obligations. The availability of these data may be subject to additional restrictions or requirements.

## Code availability

We have made the encoder code and weights available for downstream task applications. They are available via GitHub at https://github.com/SiyuanYan1/PanDerm. We have documented all experiments in detail in Methods to enable independent replication. To facilitate the broader use of our model, we have provided tutorial Jupyter notebooks and downstream evaluation code suitable for a wide scientific audience. These resources have been made available to ensure transparency and to promote further research in this field.
