# Supplementary material for: A multimodal vision foundation model for clinical dermatology
Source: Nat Med. 2025 Jun 6;31(8):2691–702. doi: 10.1038/s41591-025-03747-y (PMC12353815; doi:10.1038/s41591-025-03747-y)
Supplement: Supplementary file 1 — Supplementary Tables 1–40. [file 41591_2025_3747_MOESM1_ESM.pdf]

nature medicine

Supplementary information https://doi.org/10.1038/s41591-025-03747-y

A multimodal vision foundation model for clinical dermatology

In the format provided by the authors and unedited

Model	ISIC19b	HAM_c	BCN20000	PAD	Derm7pt_c	Dermnet	MCSI	TBP_solar	Average
CLIP_base (teacher)	88.07	96.13	91.09	91.03	77.10	89.92	99.87	94.29	90.94
CLIP_large (teacher)	89.44	96.34	93.12	92.58	80.18	90.88	99.85	95.31	92.21
MONET_large (teacher)	89.05	96.65	94.03	92.55	75.17	91.41	99.85	95.59	91.79
BIOMED_CLIP_base (teacher)	74.71	82.59	80.82	88.16	60.95	85.60	97.12	80.29	81.28
Base model	86.69	96.36	92.75	94.20	82.91	93.33	**99.88**	95.15	92.66
+ BiomedCLIP_base	86.63	97.06	93.27	91.59	82.09	90.96	98.59	95.29	91.94
+ CLIP_base	88.78	97.21	93.55	92.42	79.50	93.13	99.87	95.63	92.51
+ CLIP_large	**89.26**	**98.03**	**95.60**	**94.61**	**87.85**	**94.97**	**99.88**	**96.80**	**94.63**

Supplementary Data Table 1: **Ablation on target representation (teacher models) across various dermatology datasets**. Models include CLIP variants, MONET, BIOMED_CLIP, and PanDerm with different pretraining strategies. Metrics represent accuracy percentages. The best-performing model for each dataset is bolded and highlighted. Datasets vary in modality and size: ISIC19b, HAM_c, BCN20000 (derm, 20k/10k/12k), PAD, Derm7pt_c, Dermnet, MCSI (clinic, 2k/839/19k/400), TBP_solar (TBP, 6k). It shows that CLIP-large pretrained on the natural domain can outperform biomedical-specific CLIP (BiomedCLIP) and dermatology-specific CLIP (MONET). This can be attributed to the limited data scale of skin images in medical domain CLIP models. Thus, CLIP-large remains the best teacher model for creating target representations for masked image modeling in dermatology. When incorporating CLIP-large as the teacher model, it significantly improved the base model (+ 1.97 on average) and also outperformed the teacher model itself (+2.42 on average).

Model	HAM_c	BCN20000	PAD	Derm7pt_c	Dermnet	MCSI	TBP_solar	Average	Training time
PanDerm (FT)	**98.03**	**97.65**	93.59	**86.68**	**95.21**	98.10	**96.38**	**95.09**	$\sim$ 80 min
PanDerm (LP)	97.40	95.19	**94.50**	84.94	94.36	**99.53**	96.09	94.57	$\sim$ 5 min
Performance difference	-0.63	-2.46	+0.91	-1.74	-0.85	+1.43	-0.29	-0.52	-75 min
Modalities	derm	derm	clinic	clinic	clinic	clinic	TBP		
Size	10k	12k	2k	839	19k	400	6k		
#class	7	9	6	2	23	4	3		

Supplementary Data Table 2: **Performance comparison of PanDerm (FT) and PanDerm (LP) models across various dermatology datasets**. FT: Fine-Tuning, LP: Linear Probing. Metrics represent accuracy percentages. The best-performing model for each dataset is bolded. The performance difference row shows the change from FT to LP, with positive values indicating LP outperformed FT. Datasets vary in modality, size, and number of classes as shown in the bottom rows. It shows that PanDerm using simple linear probing can perform comparably with expensive full-parameter finetuning. This suggests that PanDerm's features are already well-suited for diverse downstream multimodal skin-related tasks without requiring further training. All models are trained and evaluated using 4 $\times$ NVIDIA RTX 6000Ada GPUs.

**Model**	**Accuracy**	**Weighted F1**
BioMedGPT (finetune)	0.7394	0.7740
BioMedGPT (Linear probe)	0.7149	0.6816
PanDerm (Finetune)	**0.8538**	**0.8538**
PanDerm (Linear probe)	0.8306	0.8252

Supplementary Data Table 3: **Performance comparison between BioMedGPT and PanDerm models on HAM10000.** Models are evaluated using both fine-tuning and linear probing (LP) approaches. Performance is reported using Accuracy and Weighted F1 score metrics. Best performance for each metric is bolded.

Dataset	Model	W_F1	AUROC	BACC	AUPR
PAD	SL_Imagenet	0.678 (0.636-0.720)***	0.887 (0.864-0.911)***	0.614 (0.540-0.688)	0.759 (0.718-0.799)***
	DINOv2	0.702 (0.662-0.743)**	0.885 (0.861-0.908)***	0.607 (0.535-0.679)*	0.753 (0.710-0.796)***
	SwaVDerm	0.656 (0.614-0.698)***	0.865 (0.838-0.891)***	0.534 (0.469-0.599)**	0.718 (0.675-0.761)***
	PanDerm	**0.768 (0.732-0.805)**	**0.935 (0.919-0.951)**	**0.694 (0.624-0.764)**	**0.849 (0.817-0.880)**
HAM10000	SL_Imagenet	0.879 (0.863-0.895)***	0.970 (0.962-0.978)***	0.653 (0.586-0.720)***	0.922 (0.909-0.936)***
	DINOv2	0.883 (0.868-0.899)***	0.964 (0.954-0.974)***	0.701 (0.637-0.765)***	0.916 (0.901-0.931)***
	SwaVDerm	0.865 (0.848-0.883)***	0.967 (0.959-0.975)***	0.592 (0.521-0.663)***	0.910 (0.896-0.924)***
	PanDerm	**0.926 (0.912-0.940)**	**0.988 (0.984-0.992)**	**0.807 (0.756-0.859)**	**0.959 (0.949-0.970)**
DermC	SL_Imagenet	0.756 (0.694-0.818)	0.797 (0.726-0.867)*	0.700 (0.631-0.770)	0.683 (0.575-0.791)*
	DINOv2	0.763 (0.702-0.824)	0.796 (0.719-0.872)**	0.717 (0.649-0.786)	0.735 (0.644-0.826)
	SwaVDerm	0.750 (0.687-0.814)	0.768 (0.690-0.846)**	0.700 (0.628-0.771)	0.658 (0.543-0.774)**
	PanDerm	**0.788 (0.728-0.847)**	**0.876 (0.824-0.928)**	**0.740 (0.672-0.808)**	**0.798 (0.710-0.886)**
BCN20000	SL_Imagenet	0.698 (0.673-0.722)***	0.914 (0.903-0.925)***	0.592 (0.537-0.647)*	0.754 (0.728-0.779)***
	DINOv2	0.724 (0.701-0.747)***	0.927 (0.917-0.938)***	0.575 (0.518-0.632)**	0.787 (0.765-0.810)***
	SwaVDerm	0.696 (0.672-0.720)***	0.908 (0.897-0.919)***	0.499 (0.444-0.554)***	0.742 (0.717-0.768)***
	PanDerm	**0.772 (0.750-0.795)**	**0.952 (0.944-0.960)**	**0.662 (0.616-0.708)**	**0.846 (0.825-0.867)**
DDI	SL_Imagenet	0.773 (0.710-0.836)	0.743 (0.639-0.847)**	0.655 (0.554-0.755)	0.412 (0.283-0.541)*
	DINOv2	0.756 (0.695-0.816)	0.749 (0.649-0.849)*	0.612 (0.518-0.706)*	0.456 (0.308-0.605)
	SwaVDerm	0.730 (0.668-0.792)	0.747 (0.650-0.845)**	0.571 (0.483-0.660)**	0.421 (0.294-0.548)*
	PanDerm	**0.790 (0.728-0.852)**	**0.843 (0.768-0.918)**	**0.722 (0.624-0.819)**	**0.551 (0.397-0.705)**
HIBA	SL_Imagenet	0.881 (0.850-0.911)**	0.881 (0.823-0.940)**	0.685 (0.612-0.759)**	0.638 (0.511-0.765)*
	DINOv2	0.884 (0.852-0.915)***	0.873 (0.816-0.930)**	0.737 (0.663-0.812)***	0.616 (0.486-0.747)*
	SwaVDerm	0.865 (0.834-0.895)***	0.854 (0.799-0.910)***	0.657 (0.585-0.729)***	0.519 (0.387-0.651)***
	PanDerm	**0.928 (0.901-0.954)**	**0.948 (0.922-0.975)**	**0.810 (0.740-0.880)**	**0.765 (0.652-0.878)**
MSKCC	SL_Imagenet	0.712 (0.691-0.732)**	0.733 (0.708-0.758)**	0.635 (0.611-0.660)	0.482 (0.444-0.521)***
	DINOv2	0.707 (0.687-0.728)**	0.722 (0.695-0.748)**	**0.662 (0.636-0.689)**	0.497 (0.457-0.537)***
	SwaVDerm	0.715 (0.696-0.733)*	0.720 (0.692-0.748)***	0.617 (0.593-0.641)**	0.515 (0.474-0.556)***
	PanDerm	**0.737 (0.718-0.756)**	**0.761 (0.735-0.787)**	0.653 (0.628-0.677)	**0.589 (0.548-0.630)**
PATCH16	SL_Imagenet	0.873 (0.867-0.878)***	0.992 (0.991-0.992)***	0.834 (0.808-0.859)***	0.936 (0.932-0.941)***
	DINOv2	0.892 (0.886-0.897)***	0.993 (0.992-0.994)**	0.820 (0.813-0.828)***	0.945 (0.941-0.949)
	SwaVDerm	0.816 (0.809-0.822)***	0.984 (0.983-0.985)***	0.742 (0.734-0.751)***	0.891 (0.885-0.896)***
	PanDerm	**0.903 (0.898-0.908)**	**0.994 (0.993-0.994)**	**0.879 (0.854-0.903)**	**0.946 (0.943-0.950)**
ISIC2024	SL_Imagenet	0.877 (0.873-0.877)***	0.849 (0.765-0.917)***	0.727 (0.635-0.811)**	0.860 (0.835-0.885)***
	DINOv2	0.851 (0.849-0.853)***	0.827 (0.745-0.870)***	0.682 (0.606-0.752)***	0.850 (0.825-0.875)***
	SwaVDerm	0.869 (0.866-0.871)***	0.852 (0.789-0.877)***	0.689 (0.599-0.774)***	0.835 (0.810-0.860)***
	PanDerm	**0.929 (0.927-0.931)**	**0.893 (0.839-0.940)**	**0.799 (0.718-0.873)**	**0.915 (0.895-0.935)**
WSI	SL_Imagenet	0.945 (0.925-0.965)	0.989 (0.977-1.002)	0.937 (0.906-0.960)***	0.941 (0.930-0.947)***
	DINOv2	0.947 (0.919-0.974)	0.988 (0.976-1.000)	0.932 (0.910-0.945)***	0.930 (0.906-0.956)***
	SwaVDerm	0.932 (0.914-0.951)	0.989 (0.978-1.000)*	0.893 (0.882-0.910)***	0.920 (0.905-0.935)***
	PanDerm	**0.953 (0.930-0.975)**	**0.994 (0.986-1.001)**	**0.976 (0.963-0.994)**	**0.981 (0.972-0.993)**

Supplementary Data Table 4: **Skin cancer diagnosis performance of different models across multinational datasets.** Models include SL_Imagenet (supervised learning on ImageNet), DINOv2, SwaVDerm, and PanDerm. Performance is reported using Weighted F1 score (W_F1), Area Under the Receiver Operating Characteristic curve (AUROC), Balanced Accuracy (BACC), and Area Under the Precision-Recall curve (AUPR). Further details on the experimental setup, datasets, and metrics are provided in **Methods**. Best-performing model for each metric and dataset is bolded and highlighted. 95% CI is included in parentheses. Significance levels for comparisons with the best model: $^{*}p < 0.05$, $^{**}p < 0.01$, $^{***}p < 0.001$. P-values calculated using a two-sided t-test.

Percent	Model	W_F1	AUROC	BACC	AUPR
5%	SL_ImageNet	0.792 (0.774-0.809)***	0.919 (0.905-0.934)***	0.337 (0.294-0.379)***	0.853 (0.838-0.867)***
	DINOv2	0.803 (0.786-0.821)***	0.928 (0.913-0.943)***	0.380 (0.322-0.437)***	0.852 (0.837-0.867)***
	SwAVDerm	0.793 (0.776-0.810)***	0.917 (0.901-0.932)***	0.322 (0.288-0.356)***	0.841 (0.826-0.855)***
	PanDerm	**0.851 (0.834-0.868)**	**0.960 (0.950-0.970)**	**0.524 (0.459-0.589)**	**0.902 (0.888-0.915)**
10%	SL_ImageNet	0.816 (0.797-0.834)***	0.937 (0.925-0.949)***	0.414 (0.365-0.464)***	0.871 (0.856-0.886)***
	DINOv2	0.824 (0.807-0.841)***	0.939 (0.926-0.953)***	0.448 (0.389-0.506)***	0.871 (0.856-0.886)***
	SwAVDerm	0.811 (0.794-0.827)***	0.923 (0.907-0.938)***	0.365 (0.329-0.402)***	0.854 (0.840-0.869)***
	PanDerm	**0.872 (0.855-0.888)**	**0.969 (0.961-0.978)**	**0.618 (0.550-0.686)**	**0.923 (0.910-0.936)**
20%	SL_ImageNet	0.838 (0.820-0.855)***	0.940 (0.928-0.952)***	0.506 (0.439-0.573)***	0.880 (0.865-0.896)***
	DINOv2	0.839 (0.822-0.856)***	0.947 (0.935-0.959)***	0.534 (0.467-0.600)**	0.884 (0.869-0.899)***
	SwAVDerm	0.825 (0.809-0.842)***	0.931 (0.916-0.946)***	0.408 (0.357-0.460)***	0.871 (0.855-0.886)***
	PanDerm	**0.889 (0.873-0.905)**	**0.975 (0.968-0.982)**	**0.665 (0.594-0.736)**	**0.935 (0.924-0.947)**
30%	SL_ImageNet	0.837 (0.820-0.855)***	0.945 (0.933-0.956)***	0.505 (0.438-0.571)***	0.883 (0.867-0.899)***
	DINOv2	0.848 (0.831-0.864)***	0.951 (0.939-0.962)***	0.559 (0.488-0.630)**	0.894 (0.880-0.909)***
	SwAVDerm	0.839 (0.822-0.857)***	0.939 (0.925-0.953)***	0.493 (0.428-0.558)***	0.879 (0.864-0.895)***
	PanDerm	**0.895 (0.880-0.911)**	**0.979 (0.973-0.985)**	**0.721 (0.653-0.788)**	**0.943 (0.931-0.954)**
50%	SL_ImageNet	0.855 (0.838-0.872)***	0.957 (0.947-0.967)***	0.565 (0.492-0.638)***	0.904 (0.889-0.919)***
	DINOv2	0.855 (0.838-0.872)***	0.953 (0.940-0.965)***	0.597 (0.529-0.664)**	0.902 (0.888-0.916)***
	SwAVDerm	0.854 (0.836-0.871)***	0.953 (0.941-0.964)***	0.557 (0.489-0.626)***	0.893 (0.878-0.908)***
	PanDerm	**0.909 (0.894-0.924)**	**0.981 (0.976-0.987)**	**0.749 (0.685-0.814)**	**0.950 (0.939-0.961)**
100%	SL_ImageNet	0.872 (0.856-0.888)***	0.967 (0.958-0.976)***	0.652 (0.584-0.720)***	0.919 (0.905-0.934)***
	DINOv2	0.876 (0.860-0.892)***	0.962 (0.951-0.972)***	0.686 (0.621-0.751)**	0.913 (0.898-0.928)***
	SwAVDerm	0.864 (0.847-0.881)***	0.963 (0.954-0.972)***	0.592 (0.520-0.664)***	0.904 (0.889-0.919)***
	PanDerm	**0.922 (0.908-0.936)**	**0.988 (0.984-0.992)**	**0.797 (0.744-0.850)**	**0.959 (0.949-0.969)**

Supplementary Data Table 5: **Label efficiency generalization performance for dermoscopic image-based skin cancer diagnosis based on HAM_clean dataset.** Metrics: W_F1 (Weighted F1), AUROC, BACC (Balanced Accuracy), AUPR (Area Under Precision-Recall Curve). The best model for each setting is bolded. 95% CI in parentheses. $^{*}p < 0.05$, $^{**}p < 0.01$, $^{***}p < 0.001$ compared to PanDerm. P-values calculated using a two-sided t-test.

Percent	Model	W_F1	AUROC	BACC	AUPR
5%	SL_ImageNet	0.543 (0.518-0.568)***	0.815 (0.799-0.831)***	0.305 (0.272-0.338)	0.567 (0.539-0.594)***
	DINOv2	0.565 (0.541-0.590)	0.840 (0.824-0.855)**	0.305 (0.278-0.332)	0.616 (0.589-0.642)
	SwAVDerm	0.541 (0.517-0.565)***	0.809 (0.793-0.825)***	0.272 (0.248-0.297)**	0.565 (0.538-0.593)***
	PanDerm	**0.586 (0.563-0.609)**	**0.859 (0.846-0.872)**	**0.304 (0.278-0.330)**	**0.628 (0.601-0.654)**
10%	SL_ImageNet	0.561 (0.536-0.586)***	0.837 (0.822-0.851)***	0.332 (0.295-0.369)***	0.599 (0.569-0.628)***
	DINOv2	0.602 (0.577-0.627)**	0.855 (0.840-0.870)***	0.373 (0.329-0.417)*	0.642 (0.614-0.671)***
	SwAVDerm	0.571 (0.546-0.596)***	0.833 (0.818-0.848)***	0.321 (0.288-0.355)***	0.590 (0.562-0.618)***
	PanDerm	**0.650 (0.626-0.675)**	**0.890 (0.877-0.903)**	**0.417 (0.374-0.459)**	**0.704 (0.678-0.731)**
20%	SL_ImageNet	0.612 (0.587-0.638)***	0.862 (0.849-0.876)***	0.392 (0.346-0.438)*	0.647 (0.619-0.675)***
	DINOv2	0.614 (0.589-0.638)***	0.872 (0.859-0.886)***	0.374 (0.330-0.418)**	0.667 (0.638-0.695)***
	SwAVDerm	0.591 (0.566-0.615)***	0.851 (0.836-0.865)***	0.357 (0.318-0.396)**	0.629 (0.601-0.656)***
	PanDerm	**0.681 (0.658-0.704)**	**0.910 (0.899-0.921)**	**0.434 (0.395-0.473)**	**0.735 (0.708-0.761)**
30%	SL_ImageNet	0.613 (0.587-0.639)***	0.866 (0.853-0.880)***	0.426 (0.373-0.478)*	0.663 (0.636-0.690)***
	DINOv2	0.648 (0.624-0.672)***	0.880 (0.867-0.894)***	0.435 (0.381-0.489)*	0.687 (0.660-0.713)***
	SwAVDerm	0.610 (0.586-0.635)***	0.861 (0.847-0.875)***	0.343 (0.315-0.372)***	0.639 (0.611-0.668)***
	PanDerm	**0.703 (0.680-0.727)**	**0.923 (0.913-0.933)**	**0.509 (0.455-0.563)**	**0.766 (0.742-0.790)**
50%	SL_ImageNet	0.649 (0.624-0.674)***	0.888 (0.875-0.901)***	0.495 (0.435-0.556)*	0.698 (0.671-0.726)***
	DINOv2	0.660 (0.636-0.684)***	0.894 (0.883-0.906)***	0.478 (0.424-0.532)*	0.720 (0.695-0.746)***
	SwAVDerm	0.628 (0.602-0.653)***	0.880 (0.867-0.893)***	0.396 (0.352-0.439)***	0.685 (0.657-0.713)***
	PanDerm	**0.733 (0.709-0.757)**	**0.939 (0.930-0.948)**	**0.578 (0.521-0.636)**	**0.801 (0.778-0.825)**
100%	SL_ImageNet	0.698 (0.673-0.723)***	0.910 (0.898-0.922)***	0.590 (0.539-0.641)	0.747 (0.722-0.773)***
	DINOv2	0.705 (0.681-0.729)***	0.923 (0.913-0.933)***	0.565 (0.506-0.624)**	0.774 (0.748-0.800)***
	SwAVDerm	0.683 (0.659-0.708)***	0.907 (0.895-0.918)***	0.479 (0.428-0.529)***	0.733 (0.707-0.758)***
	PanDerm	**0.767 (0.742-0.791)**	**0.951 (0.944-0.959)**	**0.647 (0.602-0.692)**	**0.843 (0.822-0.864)**

Supplementary Data Table 6: **Label efficiency generalization performance for dermoscopic image-based skin cancer diagnosis on BCN20000 dataset.** Metrics: W_F1 (Weighted F1), AUROC, BACC (Balanced Accuracy), AUPR (Area Under Precision-Recall Curve). The best model for each setting is bolded. 95% CI in parentheses. $^{*}p < 0.05$, $^{**}p < 0.01$, $^{***}p < 0.001$ compared to PanDerm. P-values calculated using a two-sided t-test.

Percent	Model	W_F1	AUROC	BACC	AUPR
5%	SL_ImageNet	0.826 (0.802-0.851)	0.681 (0.593-0.769)*	0.548 (0.502-0.594)	0.329 (0.214-0.444)
	DINOv2	0.825 (0.798-0.853)	0.706 (0.620-0.791)	0.553 (0.502-0.605)	0.331 (0.219-0.444)*
	SwAVDerm	0.839 (0.812-0.866)	0.660 (0.567-0.754)*	0.572 (0.518-0.626)	0.355 (0.231-0.480)
	PanDerm	**0.842 (0.811-0.874)**	**0.749 (0.663-0.834)**	**0.594 (0.534-0.654)**	**0.431 (0.299-0.562)**
10%	SL_ImageNet	**0.858 (0.829-0.888)**	0.784 (0.708-0.861)	0.622 (0.556-0.688)	0.470 (0.345-0.595)
	DINOv2	0.853 (0.822-0.884)	**0.798 (0.721-0.875)**	0.626 (0.557-0.694)	0.461 (0.322-0.600)
	SwAVDerm	0.858 (0.826-0.889)	0.732 (0.647-0.816)	**0.653 (0.581-0.725)**	0.390 (0.263-0.517)
	PanDerm	0.857 (0.826-0.888)	0.795 (0.712-0.878)	0.652 (0.583-0.721)	**0.480 (0.347-0.613)**
20%	SL_ImageNet	0.847 (0.818-0.876)	0.802 (0.739-0.865)***	0.612 (0.545-0.679)*	0.458 (0.332-0.584)
	DINOv2	**0.908 (0.879-0.937)**	0.799 (0.726-0.833)	**0.779 (0.708-0.851)**	**0.629 (0.500-0.758)**
	SwAVDerm	0.841 (0.811-0.872)*	0.798 (0.724-0.872)**	0.607 (0.541-0.673)*	0.461 (0.333-0.589)
	PanDerm	0.879 (0.848-0.910)	**0.885 (0.839-0.930)**	0.718 (0.644-0.791)	0.573 (0.443-0.703)
30%	SL_ImageNet	0.851 (0.821-0.881)	0.851 (0.794-0.908)*	0.631 (0.561-0.701)*	0.506 (0.384-0.628)
	DINOv2	0.869 (0.837-0.902)	0.854 (0.794-0.914)*	0.709 (0.638-0.781)	0.580 (0.444-0.715)
	SwAVDerm	**0.884 (0.854-0.915)**	0.814 (0.745-0.883)**	0.679 (0.609-0.748)	0.508 (0.372-0.643)
	PanDerm	0.874 (0.842-0.906)	**0.899 (0.858-0.939)**	**0.731 (0.657-0.805)**	**0.601 (0.465-0.737)**
50%	SL_ImageNet	0.872 (0.842-0.903)	0.839 (0.778-0.899)**	0.679 (0.607-0.752)	0.524 (0.399-0.650)*
	DINOv2	0.882 (0.850-0.913)	0.831 (0.766-0.896)**	0.718 (0.644-0.793)	0.540 (0.403-0.677)*
	SwAVDerm	0.873 (0.841-0.904)	0.820 (0.757-0.883)**	0.687 (0.614-0.760)	0.515 (0.382-0.648)**
	PanDerm	**0.890 (0.860-0.920)**	**0.915 (0.877-0.953)**	**0.750 (0.677-0.823)**	**0.688 (0.570-0.805)**
100%	SL_ImageNet	0.873 (0.841-0.904)*	0.878 (0.823-0.933)**	0.681 (0.610-0.751)**	0.638 (0.524-0.752)*
	DINOv2	0.879 (0.848-0.911)*	0.877 (0.820-0.934)***	0.734 (0.659-0.808)	0.631 (0.507-0.754)*
	SwAVDerm	0.860 (0.828-0.891)***	0.848 (0.791-0.905)***	0.660 (0.590-0.731)**	0.538 (0.414-0.661)***
	PanDerm	**0.912 (0.884-0.940)**	**0.949 (0.922-0.976)**	**0.774 (0.700-0.847)**	**0.771 (0.660-0.881)**

Supplementary Data Table 7: **Label efficiency generalization performance for dermoscopic image-based melanoma detection on HIBA dataset.** Metrics: W_F1 (Weighted F1), AUROC, BACC (Balanced Accuracy), AUPR (Area Under Precision-Recall Curve). The best model for each setting is bolded. 95% CI in parentheses. $^{*}p < 0.05$, $^{**}p < 0.01$, $^{***}p < 0.001$ compared to PanDerm. P-values calculated using a two-sided t-test.

Percent	Model	W_F1	AUROC	BACC	AUPR
5%	SL_ImageNet	0.693 (0.622-0.763)	0.689 (0.602-0.777)	0.630 (0.563-0.696)	0.591 (0.483-0.700)
	DINOv2	0.697 (0.632-0.762)	0.713 (0.625-0.801)	0.653 (0.580-0.726)	0.654 (0.546-0.762)
	SwAVDerm	0.589 (0.533-0.645)***	0.609 (0.519-0.700)	0.530 (0.475-0.585)***	0.478 (0.376-0.580)*
	PanDerm	**0.707 (0.642-0.772)**	**0.674 (0.587-0.762)**	**0.655 (0.585-0.725)**	**0.595 (0.495-0.695)**
10%	SL_ImageNet	0.677 (0.610-0.743)	0.693 (0.607-0.779)**	0.617 (0.551-0.683)	0.552 (0.444-0.660)*
	DINOv2	0.722 (0.655-0.788)	0.771 (0.697-0.845)	0.683 (0.611-0.755)	0.673 (0.568-0.777)
	SwAVDerm	0.609 (0.544-0.674)***	0.619 (0.526-0.712)***	0.550 (0.477-0.622)***	0.464 (0.367-0.560)***
	PanDerm	**0.738 (0.674-0.802)**	**0.785 (0.714-0.856)**	**0.682 (0.612-0.753)**	**0.666 (0.556-0.777)**
20%	SL_ImageNet	0.672 (0.610-0.734)*	0.738 (0.655-0.821)	0.608 (0.544-0.672)*	0.601 (0.487-0.716)
	DINOv2	**0.747 (0.685-0.809)**	**0.791 (0.715-0.867)**	**0.694 (0.622-0.765)**	0.662 (0.553-0.770)
	SwAVDerm	0.634 (0.572-0.697)**	0.641 (0.556-0.725)**	0.574 (0.508-0.640)**	0.489 (0.392-0.586)***
	PanDerm	0.731 (0.670-0.793)	0.765 (0.691-0.838)	0.672 (0.603-0.740)	**0.668 (0.565-0.771)**
30%	SL_ImageNet	0.714 (0.653-0.775)	0.772 (0.696-0.849)	0.654 (0.591-0.717)	0.662 (0.544-0.781)
	DINOv2	0.728 (0.663-0.794)	0.813 (0.746-0.879)	**0.681 (0.609-0.753)**	0.721 (0.629-0.813)
	SwAVDerm	0.636 (0.571-0.700)**	0.644 (0.555-0.733)***	0.577 (0.507-0.647)**	0.496 (0.390-0.602)***
	PanDerm	**0.729 (0.666-0.792)**	**0.816 (0.751-0.882)**	0.676 (0.604-0.748)	**0.722 (0.628-0.817)**
50%	SL_ImageNet	0.720 (0.655-0.785)	0.772 (0.703-0.842)	0.661 (0.593-0.730)	0.677 (0.574-0.781)
	DINOv2	0.726 (0.661-0.792)	**0.810 (0.743-0.877)**	0.675 (0.602-0.747)	**0.722 (0.630-0.813)**
	SwAVDerm	0.686 (0.617-0.755)	0.693 (0.610-0.775)**	0.635 (0.562-0.708)	0.541 (0.436-0.647)**
	PanDerm	**0.741 (0.676-0.806)**	0.804 (0.739-0.870)	**0.687 (0.616-0.758)**	0.716 (0.625-0.808)
100%	SL_ImageNet	0.754 (0.687-0.822)	0.810 (0.744-0.875)*	0.703 (0.632-0.774)	0.704 (0.595-0.813)*
	DINOv2	0.764 (0.699-0.829)	0.797 (0.723-0.870)**	0.723 (0.653-0.793)	0.733 (0.646-0.820)
	SwAVDerm	0.747 (0.686-0.809)	0.777 (0.702-0.853)**	0.696 (0.625-0.767)	0.660 (0.544-0.776)**
	PanDerm	**0.802 (0.743-0.860)**	**0.878 (0.824-0.931)**	**0.767 (0.700-0.833)**	**0.799 (0.714-0.884)**

Supplementary Data Table 8: **Label efficiency generalization performance for clinical image-based melanoma detection on DermC dataset.** Metrics: W_F1 (Weighted F1), AUROC, BACC (Balanced Accuracy), AUPR (Area Under Precision-Recall Curve). The best model for each setting is bolded. 95% CI in parentheses. $^{*}p < 0.05$, $^{**}p < 0.01$, $^{***}p < 0.001$ compared to PanDerm. P-values calculated using a two-sided t-test.

Percent	Model	W_F1	AUROC	BACC	AUPR
5%	SL_ImageNet	0.566 (0.526-0.605)	0.803 (0.775-0.832)	0.395 (0.338-0.452)	0.598 (0.555-0.641)***
	DINOv2	0.560 (0.521-0.599)*	0.804 (0.776-0.833)	0.393 (0.354-0.432)***	0.607 (0.563-0.651)***
	SwAVDerm	0.468 (0.425-0.510)***	0.695 (0.661-0.729)***	0.328 (0.279-0.378)***	0.485 (0.442-0.528)***
	PanDerm	**0.607 (0.569-0.646)**	**0.835 (0.808-0.861)**	**0.504 (0.432-0.576)**	**0.675 (0.636-0.715)**
10%	SL_ImageNet	0.580 (0.540-0.621)***	0.824 (0.795-0.852)***	0.431 (0.375-0.487)**	0.630 (0.584-0.675)***
	DINOv2	0.598 (0.557-0.638)***	0.835 (0.810-0.861)***	0.432 (0.388-0.477)***	0.652 (0.608-0.695)***
	SwAVDerm	0.515 (0.474-0.556)***	0.753 (0.721-0.785)***	0.375 (0.317-0.433)***	0.550 (0.503-0.597)***
	PanDerm	**0.673 (0.632-0.713)**	**0.877 (0.854-0.900)**	**0.560 (0.490-0.630)**	**0.733 (0.691-0.774)**
20%	SL_ImageNet	0.601 (0.561-0.640)***	0.831 (0.803-0.858)***	0.486 (0.415-0.556)*	0.661 (0.616-0.705)***
	DINOv2	0.621 (0.581-0.661)**	0.843 (0.817-0.869)***	0.472 (0.408-0.536)***	0.678 (0.634-0.723)***
	SwAVDerm	0.535 (0.494-0.576)***	0.773 (0.742-0.805)***	0.398 (0.341-0.456)***	0.579 (0.535-0.623)***
	PanDerm	**0.685 (0.645-0.726)**	**0.882 (0.860-0.904)**	**0.598 (0.526-0.670)**	**0.740 (0.699-0.781)**
30%	SL_ImageNet	0.639 (0.597-0.681)***	0.854 (0.827-0.880)***	0.493 (0.426-0.560)***	0.702 (0.657-0.747)***
	DINOv2	0.636 (0.596-0.675)***	0.855 (0.830-0.880)***	0.477 (0.412-0.543)***	0.699 (0.658-0.740)***
	SwAVDerm	0.577 (0.532-0.621)***	0.806 (0.778-0.835)***	0.461 (0.390-0.532)***	0.624 (0.578-0.671)***
	PanDerm	**0.729 (0.689-0.768)**	**0.910 (0.891-0.929)**	**0.645 (0.573-0.716)**	**0.791 (0.753-0.829)**
50%	SL_ImageNet	0.634 (0.592-0.675)***	0.861 (0.835-0.887)***	0.544 (0.472-0.615)***	0.707 (0.663-0.752)***
	DINOv2	0.656 (0.614-0.698)***	0.867 (0.840-0.893)***	0.565 (0.494-0.636)***	0.716 (0.675-0.756)***
	SwAVDerm	0.595 (0.554-0.637)***	0.826 (0.798-0.854)***	0.490 (0.422-0.558)***	0.651 (0.607-0.695)***
	PanDerm	**0.768 (0.730-0.806)**	**0.920 (0.900-0.939)**	**0.729 (0.665-0.793)**	**0.816 (0.777-0.854)**
100%	SL_ImageNet	0.658 (0.617-0.699)***	0.878 (0.854-0.902)***	0.576 (0.504-0.649)***	0.747 (0.707-0.788)***
	DINOv2	0.695 (0.653-0.737)**	0.880 (0.855-0.905)***	0.575 (0.505-0.646)***	0.747 (0.706-0.787)***
	SwAVDerm	0.664 (0.624-0.704)***	0.858 (0.833-0.882)***	0.539 (0.473-0.606)***	0.709 (0.667-0.750)***
	PanDerm	**0.758 (0.720-0.796)**	**0.931 (0.914-0.947)**	**0.710 (0.644-0.776)**	**0.844 (0.812-0.876)**

Supplementary Data Table 9: **Label efficiency generalization performance for dermoscopic image-based skin condition classification on PAD dataset.** Metrics: W_F1 (Weighted F1), AUROC, BACC (Balanced Accuracy), AUPR (Area Under Precision-Recall Curve). The best model for each setting is bolded. 95% CI in parentheses. $^{*}p < 0.05$, $^{**}p < 0.01$, $^{***}p < 0.001$ compared to PanDerm. P-values calculated using a two-sided t-test.

Percent	Model	W_F1	AUROC	BACC	AUPR
5%	SL_ImageNet	0.805 (0.799-0.812)***	0.984 (0.983-0.985)***	0.738 (0.711-0.765)***	0.886 (0.880-0.891)***
	DINOv2	0.835 (0.829-0.841)***	0.986 (0.985-0.987)***	0.756 (0.736-0.776)***	0.905 (0.900-0.909)***
	SwAVDerm	0.738 (0.731-0.745)***	0.968 (0.966-0.969)***	0.673 (0.647-0.698)***	0.806 (0.799-0.813)***
	PanDerm	**0.867 (0.861-0.872)**	**0.991 (0.991-0.992)**	**0.844 (0.836-0.853)**	**0.931 (0.927-0.935)**
10%	SL_ImageNet	0.831 (0.824-0.837)***	0.988 (0.987-0.988)***	0.789 (0.768-0.809)***	0.908 (0.903-0.913)***
	DINOv2	0.854 (0.848-0.860)***	0.989 (0.988-0.989)***	0.790 (0.764-0.816)***	0.916 (0.911-0.921)***
	SwAVDerm	0.773 (0.766-0.780)***	0.975 (0.974-0.976)***	0.704 (0.679-0.729)***	0.842 (0.835-0.849)***
	PanDerm	**0.877 (0.872-0.882)**	**0.992 (0.992-0.993)**	**0.859 (0.850-0.867)**	**0.937 (0.933-0.941)**
20%	SL_ImageNet	0.851 (0.845-0.857)***	0.990 (0.989-0.991)***	0.799 (0.772-0.826)***	0.924 (0.919-0.928)***
	DINOv2	0.869 (0.863-0.874)***	0.991 (0.990-0.991)***	0.812 (0.787-0.838)***	0.930 (0.926-0.935)***
	SwAVDerm	0.780 (0.774-0.787)***	0.977 (0.976-0.979)***	0.708 (0.687-0.729)***	0.854 (0.848-0.861)***
	PanDerm	**0.884 (0.879-0.889)**	**0.993 (0.993-0.994)**	**0.874 (0.866-0.882)**	**0.945 (0.942-0.949)**
30%	SL_ImageNet	0.862 (0.857-0.868)***	0.991 (0.990-0.991)***	0.803 (0.778-0.827)***	0.929 (0.925-0.934)***
	DINOv2	0.879 (0.873-0.884)***	0.992 (0.991-0.992)***	0.827 (0.801-0.852)*	0.937 (0.933-0.941)***
	SwAVDerm	0.793 (0.786-0.799)***	0.980 (0.978-0.981)***	0.710 (0.702-0.719)***	0.863 (0.857-0.870)***
	PanDerm	**0.896 (0.891-0.901)**	**0.994 (0.993-0.994)**	**0.866 (0.841-0.891)**	**0.947 (0.943-0.951)**
50%	SL_ImageNet	0.865 (0.859-0.871)***	0.991 (0.990-0.992)***	0.809 (0.784-0.833)***	0.931 (0.927-0.935)***
	DINOv2	0.885 (0.879-0.890)***	0.992 (0.991-0.993)***	0.821 (0.801-0.841)***	0.938 (0.934-0.942)***
	SwAVDerm	0.798 (0.792-0.805)***	0.981 (0.980-0.982)***	0.718 (0.709-0.727)***	0.871 (0.865-0.877)***
	PanDerm	**0.900 (0.895-0.904)**	**0.994 (0.993-0.995)**	**0.872 (0.848-0.897)**	**0.948 (0.944-0.951)**
100%	SL_ImageNet	0.869 (0.863-0.875)***	0.992 (0.991-0.992)***	0.829 (0.803-0.856)***	0.934 (0.930-0.938)***
	DINOv2	0.890 (0.885-0.895)***	0.993 (0.992-0.993)***	0.820 (0.813-0.828)***	0.942 (0.938-0.947)**
	SwAVDerm	–	–	–	–
	PanDerm	**0.901 (0.896-0.905)**	**0.994 (0.993-0.994)**	**0.878 (0.854-0.902)**	**0.947 (0.943-0.950)**

Supplementary Data Table 10: **Label efficiency generalization performance for fine-grained skin tumor classification on PATCH16 dataset.** Metrics: W_F1 (Weighted F1), AUROC, BACC (Balanced Accuracy), AUPR (Area Under Precision-Recall Curve). The best model for each setting is bolded. 95% CI in parentheses. $^{*}p < 0.05$, $^{**}p < 0.01$, $^{***}p < 0.001$ compared to PanDerm. SwAVDerm results for the 100% setting are missing. P-values calculated using a two-sided t-test.

Model	Dataset	W_F1	AUROC	BACC	AUPR
SL_Imagenet	MMT-09	0.661 (0.653, 0.668)***	0.860 (0.846, 0.874)***	0.404 (0.377, 0.430)***	0.482 (0.460, 0.503)***
DINOv2	MMT-09	0.672 (0.657, 0.687)***	0.858 (0.839, 0.877)***	0.433 (0.412, 0.454)***	0.474 (0.459, 0.490)***
SwaVDerm	MMT-09	0.624 (0.615, 0.634)***	0.814 (0.802, 0.825)***	0.356 (0.347, 0.365)***	0.411 (0.401, 0.422)***
PanDerm	MMT-09	**0.704 (0.699, 0.709)**	**0.901 (0.888, 0.913)**	**0.462 (0.436, 0.488)**	**0.560 (0.539, 0.581)**
SL_Imagenet	MMT-74	0.417 (0.411, 0.423)***	0.822 (0.807, 0.836)***	0.119 (0.101, 0.138)***	0.144 (0.125, 0.162)***
DINOv2	MMT-74	0.414 (0.401, 0.428)***	0.842 (0.830, 0.853)***	0.115 (0.103, 0.127)***	0.146 (0.127, 0.165)***
SwaVDerm	MMT-74	0.349 (0.340, 0.358)***	0.774 (0.758, 0.790)***	0.085 (0.073, 0.098)***	0.105 (0.085, 0.124)***
PanDerm	MMT-74	**0.488 (0.482, 0.494)**	**0.887 (0.872, 0.902)**	**0.174 (0.159, 0.189)**	**0.211 (0.186, 0.235)**
SL_Imagenet	DermNet	0.497 (0.481, 0.512)***	0.885 (0.878, 0.892)***	0.462 (0.444, 0.480)***	0.426 (0.407, 0.444)***
DINOv2	DermNet	0.536 (0.521, 0.551)***	0.902 (0.896, 0.909)***	0.505 (0.487, 0.523)***	0.476 (0.456, 0.496)***
SwaVDerm	DermNet	0.474 (0.458, 0.490)***	0.884 (0.878, 0.891)***	0.442 (0.424, 0.460)***	0.428 (0.410, 0.446)***
PanDerm	DermNet	**0.619 (0.603, 0.634)**	**0.944 (0.939, 0.949)**	**0.586 (0.568, 0.603)**	**0.623 (0.603, 0.642)**

Supplementary Data Table 11: **General multi-class skin condition classification performance of different models on MMT-09, MMT-74, and Dermnet datasets**. All models were evaluated on three datasets: MMT-09, MMT-74, and DermNet. Models include SL_Imagenet (supervised learning on ImageNet), DINOv2, SwaVDerm, and PanDerm. Performance is reported using Weighted F1 score (W_F1), Area Under the Receiver Operating Characteristic curve (AUROC), Balanced Accuracy (BACC), and Area Under the Precision-Recall curve (AUPR). The best-performing model for each metric and dataset is bolded. 95% CI is included in parentheses. $^{***}p < 0.001$ compared to PanDerm. P-values calculated using a two-sided t-test.

Dataset	Model	AUROC	Sensitivity	Specificity	BACC
DDI1P	Default	0.596 (0.567-0.624)***	0.173 (0.141-0.205)***	**0.969 (0.939-0.988)**	0.571 (0.542-0.611)***
	w/ Warp	0.673 (0.643-0.702)***	0.533 (0.493-0.562)***	0.765 (0.735-0.794)***	0.649 (0.608-0.685)***
	w/ Mask	0.648 (0.629-0.662)***	0.600 (0.571-0.626)***	0.646 (0.617-0.675)***	0.623 (0.594-0.652)***
	w/ Whole pipeline	**0.706 (0.686-0.725)**	**0.653 (0.634-0.673)**	0.741 (0.722-0.751)***	**0.697 (0.688-0.717)**
DDI2P	Default	0.683 (0.517-0.849)***	**0.940 (0.864-1.000)**	0.239 (0.115-0.593)***	0.590 (0.449-0.730)***
	w/ Warp	0.710 (0.579-0.841)***	0.942 (0.862-1.000)	0.273 (0.013-0.533)***	0.607 (0.506-0.709)***
	w/ Mask	0.695 (0.564-0.822)***	0.935 (0.853-0.995)	0.255 (0.012-0.511)***	0.595 (0.495-0.695)***
	w/ Whole pipeline	**0.767 (0.649-0.886)**	0.854 (0.797-0.911)***	**0.577 (0.387-0.768)**	**0.716 (0.621-0.810)**

Supplementary Data Table 12: **Ablation study on pre-processing methods for short-term lesion change detection based on SDDI1P and SDDI2P datasets.** Metrics: AUROC, Sensitivity, Specificity, BACC (Balanced Accuracy). Warp denoted image registration, Mask denoted lesion segmentation, and the Whole pipeline denoted our proposed pre-processing pipeline. The best model is bolded and highlighted. 95% CI in parentheses. $^{*}p < 0.05$, $^{**}p < 0.01$, $^{***}p < 0.001$. P-values calculated using a two-sided t-test.

Dataset	Model	AUROC	Sensitivity	Specificity	BACC
SDDI1	SL_Imagenet	0.616 (0.599-0.634)***	0.520 (0.501-0.543)***	0.647 (0.628-0.669)***	0.584 (0.567-0.613)***
	DINOv2	0.660 (0.649-0.678)***	0.573 (0.554-0.592)***	0.601 (0.586-0.622)***	0.587 (0.559-0.607)***
	SwaVDerm	0.632 (0.614-0.652)***	0.191 (0.163-0.214)***	**0.985 (0.961-0.999)**	0.588 (0.567-0.604)***
	PanDerm	**0.706 (0.686-0.725)**	**0.653 (0.634-0.673)**	0.741 (0.722-0.751)***	**0.697 (0.688-0.717)**
SDDI2	SL_Imagenet	0.715 (0.594-0.837)	0.870 (0.737-1.000)	0.392 (0.234-0.550)	0.631 (0.582-0.681)
	DINOv2	0.730 (0.533-0.928)	0.826 (0.673-0.979)	**0.584 (0.160-1.000)**	0.705 (0.556-0.853)
	SwaVDerm	0.656 (0.547-0.764)*	**0.970 (0.920-1.000)**	0.181 (0.051-0.412)**	0.575 (0.482-0.669)*
	PanDerm	**0.767 (0.649-0.886)**	0.854 (0.797-0.911)	0.577 (0.387-0.768)	**0.716 (0.621-0.810)**

Supplementary Data Table 13: **Short-term lesion change detection performance of different models on SDDI1 and SDDI2 datasets.** Models: SL_Imagenet, DINOv2, SwaVDerm, and PanDerm. Metrics: AUROC, Sensitivity, Specificity, BACC (Balanced Accuracy). The best model is bolded and highlighted. 95% CI in parentheses. $^{*}p < 0.05$, $^{**}p < 0.01$, $^{***}p < 0.001$. P-values calculated using a two-sided t-test.

Dataset	Model	AUROC	BACC
SDDI2M	SL_Imagenet	0.690 (0.580-0.800)**	0.588 (0.435-0.740)***
	DINOv2	0.665 (0.565-0.755)**	0.614 (0.460-0.770)***
	SwaVDerm	0.650 (0.558-0.742)**	0.540 (0.429-0.762)***
	PanDerm	**0.840 (0.769-0.911)**	**0.660 (0.472-0.848)**

Supplementary Data Table 14: **Malignant lesion change detection performance of different models on SDDI2 dataset.** classes: malignant lesion change vs others. Models: SL_Imagenet, DINOv2, SwaVDerm, and PanDerm. Metrics: AUROC and BACC (Balanced Accuracy). The best model is bolded and highlighted. 95% CI in parentheses. $^{*}p < 0.05$, $^{**}p < 0.01$, $^{***}p < 0.001$. P-values calculated using a two-sided t-test.

Dataset	Model	W_F1	AUROC	BACC	AUPR
Combinemel (2 classes)	SL_Imagenet	0.839 (0.781-0.897)	0.938 (0.898-0.978)	0.833 (0.767-0.899)	0.915 (0.867-0.964)
	DINOv2	0.841 (0.780-0.901)*	0.921 (0.872-0.971)**	0.842 (0.778-0.907)	0.886 (0.823-0.949)***
	SwaVDerm	0.847 (0.790-0.904)	0.944 (0.905-0.983)	0.858 (0.796-0.920)	0.924 (0.876-0.962)
	PanDerm	**0.889 (0.837-0.941)**	**0.964 (0.937-0.991)**	**0.882 (0.822-0.942)**	**0.944 (0.907-0.982)**
Combinemel (3 classes)	SL_Imagenet	0.661 (0.598-0.725)*	0.834 (0.789-0.879)**	0.526 (0.429-0.624)	0.734 (0.694-0.773)*
	DINOv2	0.632 (0.571-0.692)*	0.833 (0.785-0.880)**	0.474 (0.376-0.571)**	0.728 (0.689-0.767)**
	SwaVDerm	0.693 (0.633-0.744)*	0.875 (0.832-0.917)*	0.601 (0.505-0.697)	0.775 (0.728-0.822)
	PanDerm	**0.721 (0.662-0.780)**	**0.896 (0.860-0.932)**	**0.624 (0.530-0.719)**	**0.792 (0.746-0.838)**

Supplementary Data Table 15: **Metastasis prediction performance of different models on Combinemel dataset (2 classes and 3 classes)**. 2 classes: metastasis vs control (no metastasis). 3 classes: local metastasis vs distant metastasis vs control. Models include SL_Imagenet (supervised learning on ImageNet), DINOv2, SwaVDerm, and PanDerm. Metrics: Weighted F1 score (W_F1), Area Under the Receiver Operating Characteristic curve (AUROC), Balanced Accuracy (BACC), and Area Under the Precision-Recall curve (AUPR). The best-performing model for each metric and dataset is bolded and highlighted. 95% CI in parentheses. Significance levels: $^{*}p < 0.05$, $^{**}p < 0.01$, $^{***}p < 0.001$. P-values calculated using a two-sided t-test.

Model	Metric	3-year	5-year	7-year
PanDerm_multi	AUC	0.9463 (0.9004-0.9922)	**0.9462 (0.9125-0.9799)**	**0.9303 (0.8953-0.9654)**
	p-value	0.0000	0.0000	0.0000
Multi-clinical variables	AUC	0.8819 (0.7960-0.9279)	0.9018 (0.8016-0.9420)	0.8587 (0.7997-0.9177)
	p-value	0.1601	0.1521	0.1475
Single clinical variables	AUC	0.8396 (0.7999-0.8793)	0.8473 (0.7859-0.9086)	0.8021 (0.7235-0.9007)
	p-value	0.0788	0.0225	0.0480
PanDerm_single	AUC	**0.9501 (0.9095-0.9908)**	0.9312 (0.8868-0.9755)	0.9087 (0.8803-0.9371)
	p-value	0.4127	0.0871	0.0756

Supplementary Data Table 16: **Survival analysis performance comparison between PanDerm and clinical variables on CombinMel dataset**. Methods include PanDerm_multi, Multi-clinical variables, Single clinical variables, and PanDerm_single. Metrics: Area Under the Time-dependent ROC Curve (AUC) at 3, 5, and 7 years with corresponding p-values. The best-performing model for each time point is bolded. 95% CI in parentheses. P-values calculated using a two-sided t-test.

Model	3-year AUC	5-year AUC	7-year AUC
PanDerm	**0.9501 (0.9095-0.9908)**<br>p = 0.0000	**0.9312 (0.8868-0.9755)**<br>p = 0.0000	**0.9087 (0.8803-0.9371)**<br>p = 0.0000
DINOv2	0.9276 (0.8903-0.9650)<br>p = 0.2901	0.9014 (0.8273-0.9754)<br>p = 0.3659	0.8836 (0.7981-0.9691)<br>p = 0.4613
SwavDerm	0.9234 (0.8715-0.9754)<br>p = 0.2932	0.8824 (0.7943-0.9704)<br>p = 0.2066	0.8664 (0.7776-0.9552)<br>p = 0.2432
SL_ImageNet	0.9195 (0.8502-0.9889)<br>p = 0.3215	0.8858 (0.7977-0.9738)<br>p = 0.2370	0.8785 (0.7824-0.9747)<br>p = 0.4280

Supplementary Data Table 17: **Survival analysis performance comparison of different models on CombinMel dataset**. Models include PanDerm, DINOv2, SwavDerm, and SL_ImageNet. Metrics: Area Under the Time-dependent ROC Curve (AUC) at 3, 5, and 7 years with corresponding p-values. The best-performing model for each time point is bolded. 95% CI in parentheses. P-values calculated using a two-sided t-test.

Model	Prediction head	Benign			Malignant		
		Precision	Recall	F1-score	Precision	Recall	F1-score
SL_Imagenet	UD	0.999	0.943	0.971	0.007	0.464	0.015
	CLS	1.000	0.955	0.977	0.013	0.643	0.025
	CMB	1.000	0.921	0.959	0.008	0.679	0.015
	CMB_ML	1.000	0.883	0.938	0.006	0.821	0.013
DINOv2	UD	1.000	0.967	0.983	0.014	0.500	0.027
	CLS	1.000	0.954	0.977	0.013	0.679	0.026
	CMB	1.000	0.939	0.968	0.010	0.679	0.020
	CMB_ML	1.000	0.900	0.947	0.007	0.821	0.015
SwavDerm	UD	0.999	0.958	0.978	0.009	0.393	0.017
	CLS	1.000	0.961	0.980	0.012	0.500	0.023
	CMB	1.000	0.936	0.967	0.008	0.571	0.016
	CMB_ML	1.000	0.896	0.945	0.007	0.857	0.015
PanDerm	UD	1.000	0.943	0.971	0.009	0.571	0.018
	CLS	1.000	0.971	0.985	0.016	0.500	0.031
	CMB	1.000	0.928	0.962	0.009	0.714	0.018
	CMB_ML	1.000	0.887	0.940	0.007	**0.893**	0.014

Supplementary Data Table 18: **TBP-based Malignant lesion screening performance of different models and prediction head types on HOP&MYM dataset**. Maligant recall is the most crucial metrics. Results are shown for both benign and malignant classifications, including precision, recall, and F1-score.

Dataset	Model	W_F1	AUROC	BACC	AUPR
Solardamage	SL_Imagenet	0.890 (0.873-0.907)	0.950 (0.939-0.962)	0.829 (0.799-0.860)	0.899 (0.874-0.924)
	DINOv2	0.868 (0.849-0.888)	0.947 (0.935-0.958)	0.810 (0.779-0.840)	0.875 (0.847-0.903)
	SwaVDerm	0.888 (0.870-0.905)	0.958 (0.948-0.968)	0.826 (0.795-0.857)	0.908 (0.886-0.931)
	PanDerm	**0.896 (0.879-0.913)**	**0.961 (0.951-0.971)**	**0.845 (0.816-0.874)**	**0.917 (0.893-0.940)**

Supplementary Data Table 19: **Solar damage risk assessment performance of different models on HOP&MYM_solar dataset**. 3 classes: low vs medium vs high risk. Models include SL_Imagenet (supervised learning on ImageNet), DINOv2, SwaVDerm, and PanDerm. Metrics: Weighted F1 score (W_F1), Area Under the Receiver Operating Characteristic curve (AUROC), Balanced Accuracy (BACC), and Area Under the Precision-Recall curve (AUPR). The best-performing model for each metric is bolded and highlighted. 95% CI in parentheses. P-values calculated using a two-sided t-test.

Dataset	Model	W_F1	AUROC	BACC	AUPR
MYM	SL_Imagenet	0.952 (0.950-0.954)***	0.979 (0.973-0.985)***	0.810 (0.803-0.817)***	0.836 (0.821-0.850)***
	DINOv2	**0.956 (0.953-0.959)**	0.980 (0.977-0.983)***	**0.838 (0.825-0.851)*****	0.834 (0.808-0.861)***
	SwaVDerm	0.944 (0.938-0.950)***	0.973 (0.969-0.978)***	0.774 (0.765-0.782)***	0.819 (0.799-0.839)***
	PanDerm	0.956 (0.953-0.958)	**0.983 (0.979-0.987)**	0.820 (0.799-0.842)	**0.844 (0.820-0.868)**

Supplementary Data Table 20: **Nevus counting performance of different models on a subset of MYM dataset**. Models include SL_Imagenet (supervised learning on ImageNet), DINOv2, SwaVDerm, and PanDerm. Metrics: Weighted F1 score (W_F1), Area Under the Receiver Operating Characteristic curve (AUROC), Balanced Accuracy (BACC), and Area Under the Precision-Recall curve (AUPR). The best-performing model for each metric is bolded and highlighted. 95% CI in parentheses. $^{***}p < 0.001$ compared to PanDerm. P-values calculated using a two-sided t-test.

Model	AUC	W-F1	BACC
SL-Imagenet	68.09 (67.40-68.80)***	71.78 (71.27-72.33)***	62.09 (61.56-62.59)***
DINOv2	67.83 (66.69-68.03)***	69.83 (68.92-70.34)***	59.40 (58.75-59.92)***
SwavDerm	66.74 (66.22-67.10)***	68.15 (67.76-68.59)***	61.01 (60.53-61.49)***
PanDerm	**70.47 (69.76-71.15)**	**73.63 (62.74-73.92)**	**65.72 (65.13-66.28)**

Supplementary Data Table 21: **Risk stratification performance of different models on HOP&MYM datasets**. The table shows the performance metrics for different models. Metrics include Area Under the Curve (AUC), Weighted F1 score (W-F1), and Balanced Accuracy (BACC). The best-performing model for each metric is bolded and highlighted. 95% CI in parentheses. All p-values < 0.001 (***). P-values calculated using a two-sided t-test.

Dataset	Model	DSC	JAC
ISIC2018	MedSAM	0.904 (0.900-0.911)	0.841 (0.836-0.848)
	PanDerm	**0.910 (0.907-0.913)***	**0.846 (0.842-0.850)***
HAM10000	MedSAM	0.949 (0.948-0.951)	0.905 (0.904-0.907)
	PanDerm	**0.949 (0.949-0.950)**	**0.910 (0.908-0.917)**

Supplementary Data Table 22: **Lesion segmentation performance comparison of PanDerm and MedSAM on ISIC2018 and HAM10000 datasets**. Metrics: Dice Similarity Coefficient (DSC) and Jaccard Index (JAC). The best-performing model for each metric is bolded and highlighted. 95% CI in parentheses. Significance levels: $^{*}p < 0.05$, $^{**}p < 0.01$, $^{***}p < 0.001$. For ISIC2018: DSC p-value = 0.017, JAC p-value = 0.025. For HAM10000: DSC p-value = 0.793, JAC p-value = 0.112. P-values calculated using a two-sided t-test.

GPU	Model	Dataset	Training Time	Inference Time
A6000	PanDerm	ISIC2018	2h 11min	52s
		HAM10000	6h 29min	59s
	MedSAM	ISIC2018	8h 32min	2m 8s
		HAM10000	28h 57min	4m 4s
RTX3090	PanDerm	ISIC2018	2h 33min	46s
		HAM10000	7h 48min	1m 6s
	MedSAM	ISIC2018	11h 58min	2m 16s
		HAM10000	38h 23min	4m 22s

Supplementary Data Table 23: **Training and inference times comparison between PanDerm and MedSAM on lesion segmentation**. The table shows the training and inference times for PanDerm and MedSAM on ISIC2018 and HAM10000 datasets, using A6000 and RTX3090 GPUs.

Dataset	Model	DSC	JAC
ISIC2018	SL-Imagenet	0.876 (0.870-0.887)***	0.807 (0.799-0.822)***
	autoSMIM	0.848 (0.845-0.851)***	0.769 (0.766-0.771)***
	BATFormer	0.884 (0.880-0.889)***	0.815 (0.809-0.823)***
	PanDerm	**0.910 (0.907-0.913)**	**0.846 (0.842-0.850)**

Supplementary Data Table 24: **Lesion segmentation performance of different models on ISIC2018 dataset**. Models include SL-Imagenet, autoSMIM, BATFormer, and PanDerm. Metrics: Dice Similarity Coefficient (DSC) and Jaccard Index (JAC). The best-performing model for each metric is bolded and highlighted. 95% CI in parentheses. Significance levels: $^{*}p < 0.05$, $^{**}p < 0.01$, $^{***}p < 0.001$. P-values calculated using a two-sided t-test.

Dataset	Model	DSC	JAC
HAM10000	SL-Imagenet	0.927 (0.926-0.929)***	0.875 (0.873-0.878)***
	autoSMIM	0.920 (0.920-0.921)***	0.865 (0.864-0.866)***
	BATFormer	0.937 (0.935-0.939)***	0.891 (0.889-0.893)***
	PanDerm	**0.949 (0.949-0.950)**	**0.910 (0.908-0.917)**

Supplementary Data Table 25: **Lesion segmentation performance of different models on HAM10000 dataset**. Models include SL-Imagenet, autoSMIM, BATFormer, and PanDerm. Metrics: Dice Similarity Coefficient (DSC) and Jaccard Index (JAC). The best-performing model for each metric is bolded and highlighted. 95% CI in parentheses. Significance levels: $^{*}p < 0.05$, $^{**}p < 0.01$, $^{***}p < 0.001$. P-values calculated using a two-sided t-test.

Experience Group	Without AI	With AI	p-value	Corrected p-value
Low	0.64 (0.58-0.70)	**0.83 (0.78-0.88)***	0.0082	0.0246
Medium	0.67 (0.62-0.72)	**0.79 (0.75-0.83)****	<0.0001	<0.0001
High	0.78 (0.75-0.81)	**0.84 (0.82-0.86)** **	0.0390	0.1170

Supplementary Data Table 26: **Performance by experience level on human-AI collaboration study**. Comparison of accuracy with and without AI assistance for each experience group. Statistical significance was determined using paired two-sided t-tests with Bonferroni correction for multiple comparisons. 95% CI in parentheses. $^{*}p < 0.05$, $^{**}p < 0.01$, $^{***}p < 0.001$ (Bonferroni corrected) compared to performance without AI assistance.

Class	Without AI	With AI	p-value	Corrected p-value
AKIEC	0.51 (0.44-0.59)	**0.67 (0.60-0.74)***	0.0036	0.0254
BCC	0.76 (0.69-0.82)	**0.84 (0.79-0.90)**	0.0545	0.3812
BKL	0.57 (0.50-0.65)	**0.77 (0.71-0.84)****	0.0001	0.0007
DF	0.70 (0.63-0.77)	**0.81 (0.75-0.87)** *	0.0151	0.1059
MEL	0.69 (0.64-0.74)	**0.83 (0.79-0.87)****	<0.0001	0.0003
NV	0.83 (0.79-0.86)	**0.86 (0.82-0.89)**	0.1948	1.0000
VASC	0.93 (0.90-0.97)	**0.95 (0.92-0.98)**	0.4799	1.0000

Supplementary Data Table 27: **Class-specific performance comparison on human-AI collaboration study**. Comparison of accuracy with and without AI assistance for each class. Statistical significance was determined using paired two-sided t-tests with Bonferroni correction for multiple comparisons. 95% CI in parentheses. $^{*}p < 0.05$, $^{**}p < 0.01$, $^{***}p < 0.001$ compared to performance without AI assistance.

Study	Mean Recall	Recall_Mel	Recall_BCC
Tschandl et al. 2019 [?]	0.777	0.614	0.796
PanDerm	**0.804**	**0.877**	**0.860**

Supplementary Data Table 28: **Performance comparison with prior Human-AI collaboration work in HAM10000**. Comparison of model performance between PanDerm and [?] on HAM10000 dataset. PanDerm shows improvements in overall recall (+2.72%) and particularly significant gains for critical cancers like melanoma (+26.32%) and BCC (+6.42%).

Condition	Accuracy
Without AI assistance	0.69 (0.65-0.73)
With AI assistance	**0.80 (0.76-0.84)*****

Supplementary Data Table 29: **Human AI collaboration performance comparison on skin cancer classification using HAM10000 dataset**. Comparison of accuracy with and without AI assistance. 95% CI in parentheses. $^{***}p = 6.53 \times 10^{-8}$ compared to performance without AI assistance. P-values calculated using a two-sided t-test.

Dataset	Skin Tone	W_F1	Sensitivity
F17K	FST I-II (n=1195)	0.825 (0.825-0.825)	0.835 (0.835-0.835)
	FST III-IV (n=786)	0.840 (0.840-0.840)	0.851 (0.851-0.851)
	FST V-VI (n=238)	0.864 (0.864-0.864)	0.878 (0.878-0.878)
DDI	FST I-II (n=40)	0.780 (0.780-0.780)	0.750 (0.750-0.750)
	FST III-IV (n=59)	0.818 (0.818-0.818)	0.814 (0.814-0.814)
	FST V-VI (n=38)	0.854 (0.854-0.854)	0.842 (0.842-0.842)

Supplementary Data Table 30: **PanDerm performance across different skin tones on Fitzpatrick17k and DDI datasets**. The table shows the performance metrics for PanDerm on F17K and DDI datasets, stratified by Fitzpatrick Skin Type (FST) groups. Metrics include Weighted F1 score (W_F1) and Sensitivity. 95% CI in parentheses (identical to point estimate due to single measurement).

Category	Subgroup	n	W_F1	Sensitivity
Overall	All	1232	0.957	0.959
Sex	Female	563	0.963	0.965
	Male	659	0.951	0.953
Location	Face	70	0.862	0.871
	Lower extremity	240	0.968	0.971
	Abdomen	126	0.984	0.984
	Upper extremity	120	0.967	0.967
	Back	256	0.919	0.922
	Trunk	259	0.993	0.992
	Scalp	8	1.000	1.000
	Hand	18	1.000	1.000
	Unknown	40	1.000	1.000
	Chest	33	0.879	0.879
	Neck	18	1.000	1.000
	Foot	30	0.950	0.967
	Genital	8	1.000	1.000
Age	Old	517	0.923	0.927
	Medium	669	0.984	0.984
	Young	34	0.913	0.941

Supplementary Data Table 31: **Model robustness analysis across subgroups on HAM10000 dataset**. The table shows performance metrics across different subgroups based on sex, location, and age. Metrics include sample size (n), Weighted F1 score (W_F1), and Sensitivity. All metric values are point estimates.

**Characteristic**	**n (%) or Mean ± SD**
**Total Participants**	
Total	193
**Age**	
Age (years)	52.2 ± 12.0
**Sex**	
Female	82 (42.5%)
Male	111 (58.5%)
**Ancestry/Ethnicity**	
European/British	164 (85.0%)
Mixed/Other	29 (15.0%)
**Skin Phototype**	
Burns easily, tans slightly	52 (26.9%)
Burns moderately, tans gradually	123 (63.7%)
Rarely burns, tans well	18 (9.3%)
**Innate Skin Color**	
Fair/Type I	144 (74.6%)
Medium/Type II	48 (24.9%)
Olive/Type III	1 (0.5%)

Supplementary Data Table 32: Demographic characteristics of MYM cohort.

**Characteristic**	**N (%) or Mean ± SD**
**Sample Size**	
Total	314
**Age**	
Age (years)	56.1 ± 12.8
**Sex**	
Male	120 (38.2%)
Female	194 (61.8%)
**Ancestry**	
European/British	297 (94.6%)
Other/Mixed	17 (5.4%)
**Birth Place**	
Australia	268 (85.4%)
Overseas	46 (14.6%)
**Melanoma History**	
Yes	304 (96.8%)
No	10 (3.2%)
**Non-melanoma Skin Cancer**	
Yes	195 (62.1%)
No	119 (37.9%)
**Innate Skin Color**	
Fair/Type I	269 (85.7%)
Medium/Type II	44 (14.0%)
Olive/Type III	1 (0.3%)

Supplementary Data Table 33: Demographic and clinical characteristics of HOP cohort.

**Characteristic**	**N (%)**
**Total Participants**	54
**Study Sites**	
QLD 1 - PAH	26 (48.1)
NSW 3 - MIA	28 (51.9)
**Age (years)**	
Range	19-75
Mean	53.4
**Gender**	
Male	31 (57.4)
Female	23 (42.6)
**Melanoma Risk Group**	
Very High	44 (81.5)
High	8 (14.8)
Low/Average	1 (1.9)
Unknown	1 (1.9)
**Primary Diagnosis (Pathologist 1)**	
Total Naevi	37 (68.5)
Common/Dermal/Congenital	14 (25.9)
Dysplastic Compound	22 (40.7)
Dysplastic Junctional	8 (14.8)
Total Melanomas	13 (24.1)
In Situ	11 (20.4)
Thin Invasive	2 (3.7)
Other Lesions	4 (7.4)
**Number of Lesions per Patient**	
1	36 (66.7)
2	12 (22.2)
3	2 (3.7)
4	2 (3.7)
5	2 (3.7)

Supplementary Data Table 34: Demographic characteristics of the ACEMID_pilot_study dataset.

**Characteristic**	**N (%)**
**Total Participants**	1254
**Age (years)**	
Mean	47.22
Median	46
Range	11-88
**Sex**	
Male	602 (48.0)
Female	652 (52.0)
**Melanoma History**	
Personal history of melanoma	589 (47.0)
No personal history of melanoma	665 (53.0)
**Facultative Skin Colour (n=1245)**	
Fair/Type 1	635 (51.0)
Medium/Type 2	510 (41.0)
Olive/Type 3	100 (8.0)
**Self-reported Ancestry***	
British/Irish	963
West/Northern European	228
No data or other (not specified)	104
Southern European	36
Eastern European	23
Asian	18
Middle Eastern	8
Other (specified)	8
Indigenous Australian	4
Pacific Islander	4

Supplementary Data Table 35: Demographic characteristics of the NSSI dataset.
* Multiple ancestries could be reported by each participant

Hyper-parameter	Value
Teacher model	CLIP
First input size	224
Second input size	196
Second interpolation	bicubic
Number of output dimensions	768
Crop min size	0.4
Crop max size	1
Patch size	16
Vocabulary size	8000
Batch size	480
Learning rate	1.5e-3
Warmup epochs	20
Total epochs	500
Gradient clipping max norm	3.0
Layer scale init value	1e-5
Color jitter	0.4
Drop path	0.2
Mask generator	block
Number of mask patches	118
Decoder layer scale init value	1e-5
Regressor depth	4
Decoder depth	0
Decoder embed dimension	1024
Decoder number of heads	16
Align loss weight	0
Latent alignment loss weight	1
Number of GPUs	4
Distributed launch	torchrun
Processes per node	4

Supplementary Data Table 36: **PanDerm hyperparameters used in pretraining**. 4 $\times$ 80GB NVIDIA H100 GPUs were used for pretraining.

Hyperparameter	Value
Batch size	256
Epochs	50
learning rate	5e-4
Layer decay	0.75
Weight decay	0.05
Drop path	0.2
Reprob	0.25
Mixup	0.8
Cutmix	1.0

Supplementary Data Table 37: **PanDerm hyperparameters used in finetuning**. A single 49GB NVIDIA 6000Ada GPU was used for downstream finetuning.

**Major Category**	**Classes**
**Skin Cancer and High-Risk Lesions (13)**	Basal Cell Carcinoma - Nodular, Basal Cell Carcinoma - Superficial, Basal Cell Carcinoma - Pigmented, Basal Cell Carcinoma - Recurrent, Melanoma - Nodular, Lentigo Maligna, Squamous Cell Carcinoma - Common, Squamous Cell Carcinoma - Bowen's, Keratoacanthoma, Actinic Keratosis - Common, Actinic Keratosis - Hypertrophic, Actinic Keratosis - Pigmented, Actinic Cheilitis
**Melanocytic Lesions (22)**	Compound Nevus, Junctional Nevus, Dermal Nevus, Acral Parallel Pattern Nevus, Acral Lattice Pattern Nevus, Acral Untyped Pattern Nevus, Blue Nevus, Spitz Nevus, Reed Nevus, Congenital Nevus, Halo Nevus, Agminated Nevus, Atypical Nevus, Solar Lentigo, Lentigo Simplex, Ink-spot Lentigo, Ephelides, Melanosis, En Cocarde Nevus, Lentiginous Nevus, Subungual Melanocytic Nevus, Nevus with Regression
**Common Benign Growths (13)**	Seborrheic Keratosis - Common, Seborrheic Keratosis - Inflamed, Seborrheic Keratosis - Pigmented, Dermatofibroma, Epidermal Cyst, Sebaceous Hyperplasia, Skin Tag, Fibrous Papule of Face, Cutaneous Horn, Comedone, Accessory Nipple, Myxoid Cyst, Chondrodermatitis
**Inflammatory Conditions (8)**	Psoriasis, Eczema, Dermatitis, Folliculitis, Granuloma Annulare, Lichenoid Dermatitis, Porokeratosis, Hypertrophic Lichen Planus
**Vascular and Infectious Lesions (10)**	Angiokeratoma, Angioma, Telangiectasia, Hematoma - Common, Hematoma - Subcorneal, Hematoma - Subungual, Molluscum Contagiosum, Wart, Vascular Malformation, Pyogenic Granuloma
**Others (8)**	Post-inflammatory Changes, Nail Dystrophy, Hypertrophic Scar, Atrophic Scar, Excoriation, Traumatic Changes, Striae, Chemical Burn

Supplementary Data Table 38: The taxonomy of 74 dermatological conditions covered in MMT-74 dataset.

**Characteristic**	**Melanoma N (%)**	**Benign N (%)**
**Gender**		
Male	48 (53.9)	36 (40.0)
Female	41 (46.1)	54 (60.0)
Previous melanoma	67 (75.2)	57 (63.3)
Previous NMSC	44 (49.4)	20 (22.2)
Family history of melanoma	49 (55.0)	38 (42.2)
**Naevi count**		
<20	0 (0.0)	0 (0.0)
20-50	3 (3.4)	1 (1.1)
50-100	27 (30.3)	15 (16.7)
100-200	14 (15.7)	33 (36.7)
200-500	42 (47.2)	39 (43.3)
>500	3 (3.4)	2 (2.2)
**Atypical Naevi count**		
<5	19 (21.3)	22 (24.4)
5-15	28 (31.5)	29 (32.2)
>15	42 (47.2)	39 (43.0)
**Fitzpatrick Skin Phototype**		
I	12 (13.4)	11 (12.2)
II	42 (47.1)	47 (52.2)
III	28 (31.4)	32 (35.6)
IV	0 (0.0)	0 (0.0)
V	7 (7.8)	0 (0.0)
**Location**		
Head and neck	7 (7.8)	7 (7.8)
Chest	9 (10.1)	9 (10.0)
Abdomen	7 (7.8)	14 (15.6)
Back	21 (23.5)	23 (25.6)
Upper limb	19 (21.3)	13 (14.4)
Lower limb	26 (29.2)	24 (26.7)
**Histopathology (Melanoma)**		
In situ unspecified	16 (18.0)	–
In situ SSM	28 (31.4)	–
Lentigo maligna	11 (12.3)	–
Invasive SSM	32 (36.0)	–
Invasive LMM	2 (2.2)	–
**Histopathology (Benign)**		
Dysplastic naevus	–	36 (40.0)
Compound naevus	–	25 (27.8)
Junctional naevus	–	17 (18.9)
Intradermal naevus	–	8 (8.9)
Other	–	4 (4.4)

Supplementary Data Table 39: Demographic characteristics of the SDDI_Alfred dataset.

Characteristic	N (%)
**Age**	
$>$ 60 years	190 (51.4)
$\leq$ 60 years	180 (48.6)
**Sex**	
Female	172 (47.3)
Male	198 (53.5)
**Stage at Diagnosis**	
Stage I	261 (70.5)
Stage II	45 (12.2)
Stage III	61 (16.5)
Stage IV	3 (0.8)
**T Classification**	
T1a	193 (59.2)
T1b	28 (8.6)
T2a	56 (18.6)
T2b	13 (4.0)
T3a	21 (6.4)
T3b	11 (3.4)
T4a	7 (2.1)
T4b	42 (13.2)
Unknown	1 (0.3)
**SLNB Status**	
Not Performed	265 (71.6)
Positive	40 (10.8)
Negative	65 (17.6)
**N Classification**	
N1	40 (10.8)
N2	14 (3.8)
N3	7 (1.8)
Unknown	2 (0.5)

Supplementary Data Table 40: Demographic characteristics of the CombinMel dataset.
