# Supplementary material for: A multimodal vision foundation model for clinical dermatology
Source: Nat Med. 2025 Jun 6;31(8):2691–702. doi: 10.1038/s41591-025-03747-y (PMC12353815; doi:10.1038/s41591-025-03747-y)
Supplement: Supplementary file 2 — Reporting Summary [file 41591_2025_3747_MOESM2_ESM.pdf]

nature portfolio

Corresponding author(s): Zongyuan Ge

Last updated by author(s): Mar 30, 2025

Reporting Summary

Nature Portfolio wishes to improve the reproducibility of the work that we publish. This form provides structure for consistency and transparency in reporting. For further information on Nature Portfolio policies, see our Editorial Policies and the Editorial Policy Checklist.

Statistics

For all statistical analyses, confirm that the following items are present in the figure legend, table legend, main text, or Methods section.

n/a	Confirmed	
☐	☒	The exact sample size (*n*) for each experimental group/condition, given as a discrete number and unit of measurement
☐	☒	A statement on whether measurements were taken from distinct samples or whether the same sample was measured repeatedly
☐	☒	The statistical test(s) used AND whether they are one- or two-sided<br>*Only common tests should be described solely by name; describe more complex techniques in the Methods section.*
☒	☐	A description of all covariates tested
☒	☐	A description of any assumptions or corrections, such as tests of normality and adjustment for multiple comparisons
☐	☒	A full description of the statistical parameters including central tendency (e.g. means) or other basic estimates (e.g. regression coefficient) AND variation (e.g. standard deviation) or associated estimates of uncertainty (e.g. confidence intervals)
☐	☒	For null hypothesis testing, the test statistic (e.g. *F*, *t*, *r*) with confidence intervals, effect sizes, degrees of freedom and *P* value noted<br>*Give P values as exact values whenever suitable.*
☒	☐	For Bayesian analysis, information on the choice of priors and Markov chain Monte Carlo settings
☒	☐	For hierarchical and complex designs, identification of the appropriate level for tests and full reporting of outcomes
☒	☐	Estimates of effect sizes (e.g. Cohen's *d*, Pearson's *r*), indicating how they were calculated

Our web collection on statistics for biologists contains articles on many of the points above.

Software and code

Policy information about availability of computer code

Data collection	Scripts for data collection and processing were written in Python (version 3.9.19) using the libraries Pandas (version 2.2.2), Numpy (version 1.26.4), and Pillow (version 10.3.0).
Data analysis	For self-supervised pretraining, we used 4 x 80GB NVIDIA H100 GPUs configured for multi-GPU single node training using DistributedDataParallel (DDP) as implemented by Python (v.3.9.13), PyTorch (v.2.2.1, CUDA 11.8) and Torchvision (v.0.17.1). The CAE-v2 code is used as the codebase to develop our foundation model, which can be found in its official repository (https://github.com/Atten4Vis/CAE). For downstream task evaluation, all experiments were conducted on 4 x 49 GB NVIDIA 6000 Ada GPUs. We used Python (v.3.9.19), PyTorch (v.2.2.2, CUDA 11.8), and Torchvision (v.0.17.2) for finetuning tasks, and Python (v.3.10.14), PyTorch (v.2.2.2, CUDA 11.8) and Torchvision (v.0.17.2) for linear probing tasks. We used Scikit-learn (v1.2.1) for logistic regression in the linear probing setting. Implementation of other comparative pretrained models was modified based on the official configuration in their respective repositories: MAE (https://github.com/facebookresearch/mae) , SL ImageNet (https://huggingface.co/timm/vit_large_patch16_224.orig_in21k), DINOv2 (https://github.com/facebookresearch/dinov2) , SwAVDerm (https://github.com/shenyue-98/SwAVDerm) , autoSMIM (https://github.com/Wzhjerry/autoSMIM) , BATFormer (https://github.com/xianlin7/BATFormer) , MedSAM (https://github.com/bowang-lab/MedSAM) , ResNet50 (https://pytorch.org/vision/main/models/generated/torchvision.models.resnet50.html) , MILAN (https://github.com/zejiangh/MILAN) , CLIP (https://github.com/openai/CLIP) , BiomedCLIP (https://huggingface.co/microsoft/BiomedCLIP-PubMedBERT_256-vit_base_patch16_224) , and MONET (V1.0) (https://github.com/suinleelab/MONET/tree/main). To facilitate the broader use of our model, we have provided tutorial using Jupyter notebooks. The downstream evaluation code and model weights are available at https://github.com/SiyuanYan1/PanDerm.

For manuscripts utilizing custom algorithms or software that are central to the research but not yet described in published literature, software must be made available to editors and reviewers. We strongly encourage code deposition in a community repository (e.g. GitHub). See the Nature Portfolio guidelines for submitting code & software for further information.

Data

Policy information about availability of data

All manuscripts must include a data availability statement. This statement should provide the following information, where applicable:

- Accession codes, unique identifiers, or web links for publicly available datasets
- A description of any restrictions on data availability
- For clinical datasets or third party data, please ensure that the statement adheres to our policy

Most datasets used in this study are publicly available. These datasets used for skin lesion diagnosis and segmentation tasks can be accessed through various repositories. The ISIC archive (https://www.isic-archive.com/) hosts several datasets, including MSKCC and HIBA. Other widely used benchmark datasets are available through their respective portals: BCN20000 (https://figshare.com/ articles/journal contribution/BCN20000 Dermoscopic Lesions in the Wild/24140028/1), PAD-UFES-20 (https://www.kaggle.com/datasets/mahdavi1202/skin-cancer), DDI (https://ddi-dataset.github.io/index.html), Derm7pt (https://derm.cs.sfu.ca/Welcome.html), ISIC2024 (https://www.kaggle.com/competitions/isic-2024challenge), Med-Node (https://www.kaggle.com/datasets/prabhavsanga/med-node), DermNet (https://www. kaggle.com/datasets/shubhamgoel27/dermnet), WSI (https://portal.gdc.cancer.gov/projects/TCGA-SKCM), PATCH16 (https://heidata.uni-heidelberg.de/dataset.xhtml?persistentId=doi:10.11588/data/7QCR8S), ISIC2018 task1 and HAM10000 (https://challenge.isic-archive.com/data/), SDDI1 (https://api.isicarchive.com/collections/328/), PH2 (https://www.fc.up.pt/addi/ph2%20database.html), and SD-128 (https://huggingface.co/datasets/resyhgerwshshgdfghsdfgh/SD-198). Access to in-house datasets is restricted due to patient privacy considerations. These include MMT for dermoscopic and clinical image pretraining and downstream multi-skin condition classification, NSSI for sequential dermoscopic image pretraining, ACEMID path for dermatopathology pretraining, Edu1 and Edu2 for clinical image pretraining, SDDI2 for lesion change detection, SDDI Alfred for reader study 1 (early-melanoma detection), and the TBP data from MYM and HOP studies for all TBP-based pretraining and evaluation. Researchers interested in accessing these datasets should direct their requests to the corresponding author. All requests will receive a response within two weeks of submission. Requests will be evaluated according to institutional and departmental policies to ensure compliance with intellectual property rights and patient privacy obligations. The availability of these data may be subject to additional restrictions or requirements.

Research involving human participants, their data, or biological material

Policy information about studies with human participants or human data. See also policy information about sex, gender (identity/presentation), and sexual orientation and race, ethnicity and racism.

Reporting on sex and gender	Biological sex information for HAM10000 dataset was considered for model robustness experiments. Experiments were conducted both on female and male (detail in Extended Data Table 22).
Reporting on race, ethnicity, or other socially relevant groupings	We examine diagnostic accuracy across images of different skin tones based on the Fitzpatrick Skin Type scale, utilizing the public Fitzpatrick17k (https://github.com/mattgroh/fitzpatrick17k) and DDI datasets (https://stanfordaimi.azurewebsites.net/datasets/35866158-8196-48d8-87bf-50dca81df965) to evaluate our foundation model's performance across different skin tones.
Population characteristics	Population characteristics used in this study are available in Supplementary Table 32-35 and 39-40.
Recruitment	No patient recruitment was necessary for using these dermatological images retrospectively
Ethics oversight	MYM study was approved by the Metro South Health Human Research Ethics Committee on 21 April 2016 (approval number: HREC/16/QPAH/125). Ethics approval has also been obtained from the University of Queensland Human Research Ethics Committee (approval number: 2016000554), Queensland University of Technology Human Research Ethics Committee (approval number: 1600000515) and QIMR Berghofer (approval number: P2271).<br>The HOP study has received Human Research Ethics Committee (HREC) approval from Metro South Health HREC (HREC/17/QPAH/816) and The University of Queensland HREC (2018000074).<br>The ComBineMel dataset is part of the Computer biomarkers evaluation of invasive melanoma (ComBine Mel) study. The study was approved by the Alfred Hospital Ethics Committee on 08 August 2023 (approval number: HREC/98200/Alfred-2023). The study follows the National Statement on Ethical Conduct in Human Research (2007) protocols.<br>SDDI2 dataset is approved by the Ethics Review Board of the Medical University of Vienna.<br>MMT data study is part of a research agreement study with Monash eResearch Centre and was approved through the Monash University Human Research Ethics Committee (MUHREC).<br>The NSSI dataset is part of the Brisbane Naevus Morphology Study, circa 2009-2014. The study followed the Declaration of Helsinki protocols and was approved by the Princess Alexandra Hospital human research ethics committee. The ACEMID_path study has received approval from the Alfred Hospital Ethics Committee (approval number: 746/23) to share data accrued for registered trial ACTRN12619001706167 (ACEMID) under the Metro South Human Research Committee protocol HREC/2019/QMS/57206 and the University of Queensland Human Research Ethics Committee protocol 2019003077. The SDDI_Alfred study has received approval from the Alfred Hospital Ethics Committee (approval number: 198/19) for use of sequential dermoscopic imaging data. Only de-identified retrospective data was used for research, without the active involvement of patients.

Note that full information on the approval of the study protocol must also be provided in the manuscript.

Field-specific reporting

Please select the one below that is the best fit for your research. If you are not sure, read the appropriate sections before making your selection.

☒ Life sciences ☐ Behavioural & social sciences ☐ Ecological, evolutionary & environmental sciences

Life sciences study design

All studies must disclose on these points even when the disclosure is negative.

Sample size

Our sample size was determined based on the availability of high-quality multimodal dermatological data collected from our collaborating hospitals, universities, and select public datasets. We did not pre-define the sample size as our goal was to maximize foundation model performance by incorporating the largest possible training dataset.

For pre-training, we utilized a comprehensive collection of images across multiple modalities: 405,856 TBP titles from HOP and MYM cohorts and 352,034 TBP tiles from the public ISIC2024 dataset; 316,499 dermoscopic images from the MMT dataset, 38,110 from HOP and MYM cohorts, and 29,832 from the NSSI dataset; 310,951 clinical images from MMT, 81,947 from Edu1, and 67,430 from Edu2; and 377,764 dermatopathology tiles from TCGA-SKCM, 88,971 from UAH89K, and 80,312 from ACEMID.

Given that most prior self-supervised dermatological models were trained on significantly smaller web datasets (2-20 times smaller), we anticipated that our collection of over 2 million high-quality multimodal data points from real-world would be sufficient to train an effective dermatology foundation model. For information about downstream datasets, please refer to the 'Datasets and Evaluation' subsection in the Methods section of the manuscript.

Data exclusions

For pre-training data curation, we excluded sensitive data that could potentially identify individuals, such as human faces, as well as low-quality images containing a high proportion of irrelevant background elements like dense hair, clothing, or extraneous environmental details.

Replication

Replication attempts using our code successfully reproduced the reported model results, validating the reliability and reproducibility of our findings. Code is available at https://github.com/SiyuanYan1/PanDerm

Randomization

For downstream evaluations requiring train, validation, and test splits, we utilized official splits provided by the original dataset creators when available. In cases where official splits were not provided, we created random splits. To prevent data leakage, we generally implemented patient-level stratified randomization when possible, ensuring that data from the same patient remained within a single split.

Blinding

When randomly assigning patients to training, validation, and testing groups, investigators were blinded to patient covariates and all dataset features not essential for conducting the research. For reader studies, raters were not allocated into experimental groups. Images were randomly selected according to the protocol described in the Methods section.

Reporting for specific materials, systems and methods

We require information from authors about some types of materials, experimental systems and methods used in many studies. Here, indicate whether each material, system or method listed is relevant to your study. If you are not sure if a list item applies to your research, read the appropriate section before selecting a response.

Materials & experimental systems

n/a	Involved in the study	
☒	☐	Antibodies
☒	☐	Eukaryotic cell lines
☒	☐	Palaeontology and archaeology
☒	☐	Animals and other organisms
☒	☐	Clinical data
☒	☐	Dual use research of concern
☒	☐	Plants

Methods

n/a	Involved in the study	
☒	☐	ChIP-seq
☒	☐	Flow cytometry
☒	☐	MRI-based neuroimaging

Plants

Seed stocks

N/A

Novel plant genotypes

N/A

Authentication

N/A
